# Impaired fatty acid metabolism perpetuates lipotoxicity along the transition to chronic kidney injury

**DOI:** 10.1172/jci.insight.161783

**Published:** 2022-09-22

**Authors:** Anna Rinaldi, Hélène Lazareth, Virginie Poindessous, Ivan Nemazanyy, Julio L. Sampaio, Daniele Malpetti, Yohan Bignon, Maarten Naesens, Marion Rabant, Dany Anglicheau, Pietro E. Cippà, Nicolas Pallet

**Affiliations:** 1 Department of Medicine, Division of Nephrology, Ente Ospedaliero Cantonale, Lugano, Switzerland.; 2 Laboratories for Translational Research, Ente Ospedaliero Cantonale, Bellinzona, Switzerland.; 3 Université Paris Cité, INSERM UMRS1138, Centre de Recherche des Cordeliers, Paris, France.; 4 PMM: The Metabolism-Metabolome Platform, Necker Federative Research Structure, INSERM US24/CNRS UMS3633, Paris, France.; 5 CurieCoreTech Metabolomics and Lipidomics Technology Platform, Institut Curie, Paris, France.; 6 Istituto Dalle Molle di Studi sull’Intelligenza Artificiale (IDSIA), USI/SUPSI, Lugano, Switzerland.; 7 Department of Biomedical Sciences, University of Lausanne, Lausanne, Switzerland.; 8 Department of Microbiology, Immunology and Transplantation, KU Leuven, Leuven, Belgium.; 9 Department of Pathology and; 10 Department of Nephrology and Kidney Transplantation, Assistance Publique Hôpitaux de Paris, Necker Hospital, Paris, France.; 11 Université Paris Cité, INSERM U1151, Paris, France.; 12 Department of Clinical Chemistry and; 13 Department of Nephrology, Assistance Publique Hôpitaux de Paris, Georges Pompidou European Hospital, Paris, France.

**Keywords:** Nephrology, Transplantation, Bioenergetics

## Abstract

Energy metabolism failure in proximal tubule cells (PTCs) is a hallmark of chronic kidney injury. We combined transcriptomic, metabolomic, and lipidomic approaches in experimental models and patient cohorts to investigate the molecular basis of the progression to chronic kidney allograft injury initiated by ischemia/reperfusion injury (IRI). The urinary metabolome of kidney transplant recipients with chronic allograft injury and who experienced severe IRI was substantially enriched with long chain fatty acids (FAs). We identified a renal FA-related gene signature with low levels of carnitine palmitoyltransferase 2 (Cpt2) and acyl-CoA synthetase medium chain family member 5 (Acsm5) and high levels of acyl-CoA synthetase long chain family member 4 and 5 (Acsl4 and Acsl5) associated with IRI, transition to chronic injury, and established chronic kidney disease in mouse models and kidney transplant recipients. The findings were consistent with the presence of Cpt2^–^Acsl4^+^Acsl5^+^Acsm5^–^ PTCs failing to recover from IRI as identified by single-nucleus RNA-Seq. In vitro experiments indicated that ER stress contributed to CPT2 repression, which, in turn, promoted lipids’ accumulation, drove profibrogenic epithelial phenotypic changes, and activated the unfolded protein response. ER stress through CPT2 inhibition and lipid accumulation engaged an auto-amplification loop leading to lipotoxicity and self-sustained cellular stress. Thus, IRI imprints a persistent FA metabolism disturbance in the proximal tubule, sustaining the progression to chronic kidney allograft injury.

## Introduction

Understanding the mechanisms of chronic kidney injury and identifying therapeutic targets are research priorities in renal medicine. A key contributor to chronic kidney histological damage is acute kidney injury (AKI), especially ischemia/reperfusion injury (IRI), which primarily targets proximal renal tubular cells (PTCs) (1). AKI increases the risk of chronic kidney damage several-fold even in those experiencing a fully reversible episode of AKI (2, 3), and maladaptive repair of the tubular epithelium explains the high rate of fibrogenesis in the resolution of an AKI episode (4–6). Chronic kidney allograft injury results from alloimmune and nonimmune injuries leading to graft failure. IRI occurring peri-transplantation period contributes to progressive allograft dysfunction, but the molecular understanding of the progression from acute to chronic injury is limited (7, 8). Kidney transplantation offers a unique opportunity to study the response to tissue injury in humans, since it universally begins with IRI in well-defined conditions at the time of surgery, and protocol follow-up with biopsies and urine sampling provide access to renal tissue over time.

PTCs, because of their essential functions of reabsorption and secretion of solutes, are highly energy-intensive cells and therefore dependent on ATP production, which can only be ensured if the mitochondrial energetic metabolism is preserved (9). PTCs’ energetic metabolism is strongly dependent on the availability and usage of fatty acids (FAs), which are their main source of energy, and any deregulation of FA synthesis, intracellular transport, stockage, and beta-oxidation (fatty acid oxidation, FAO) will have deleterious consequences (10–12). FA accumulation is observed after ischemic AKI, most probably because of a lack of oxidative substrates to accomplish FAO, which is shut down during oxygen deprivation (13, 14). In the last few years, the documentation of the major metabolic disturbances coexisting with chronic kidney disease (CKD) has highlighted the fact that a dysregulation in FAO affects the fate of tubular epithelial cells, by promoting inflammation and fibrogenesis (10, 15).

Cellular stress responses generated upon IRI not only impair energetic metabolism but also affect signaling pathways involved in cell differentiation, proliferation, and programmed death and participate in the transition from acute to chronic kidney injury (4–6, 13, 16–18). Whereas persistent cell-extrinsic perturbations, for example hypoxia, are well-known cell stressors contributing to the progression to CKD, the cell-intrinsic determinants involved in the maintenance of a dysfunctional cellular state over time are poorly understood. By starting from the analysis of urinary metabolites associated with chronic kidney allograft injury, we identified a persistent alteration of FA metabolism as a critical element in a carnitine palmitoyltransferase 2–dependent amplification loop contributing to CKD progression.

## Results

### Higher urinary long chain FA levels in kidney allografts progressing to chronic allograft injury.

**Figure 1:**
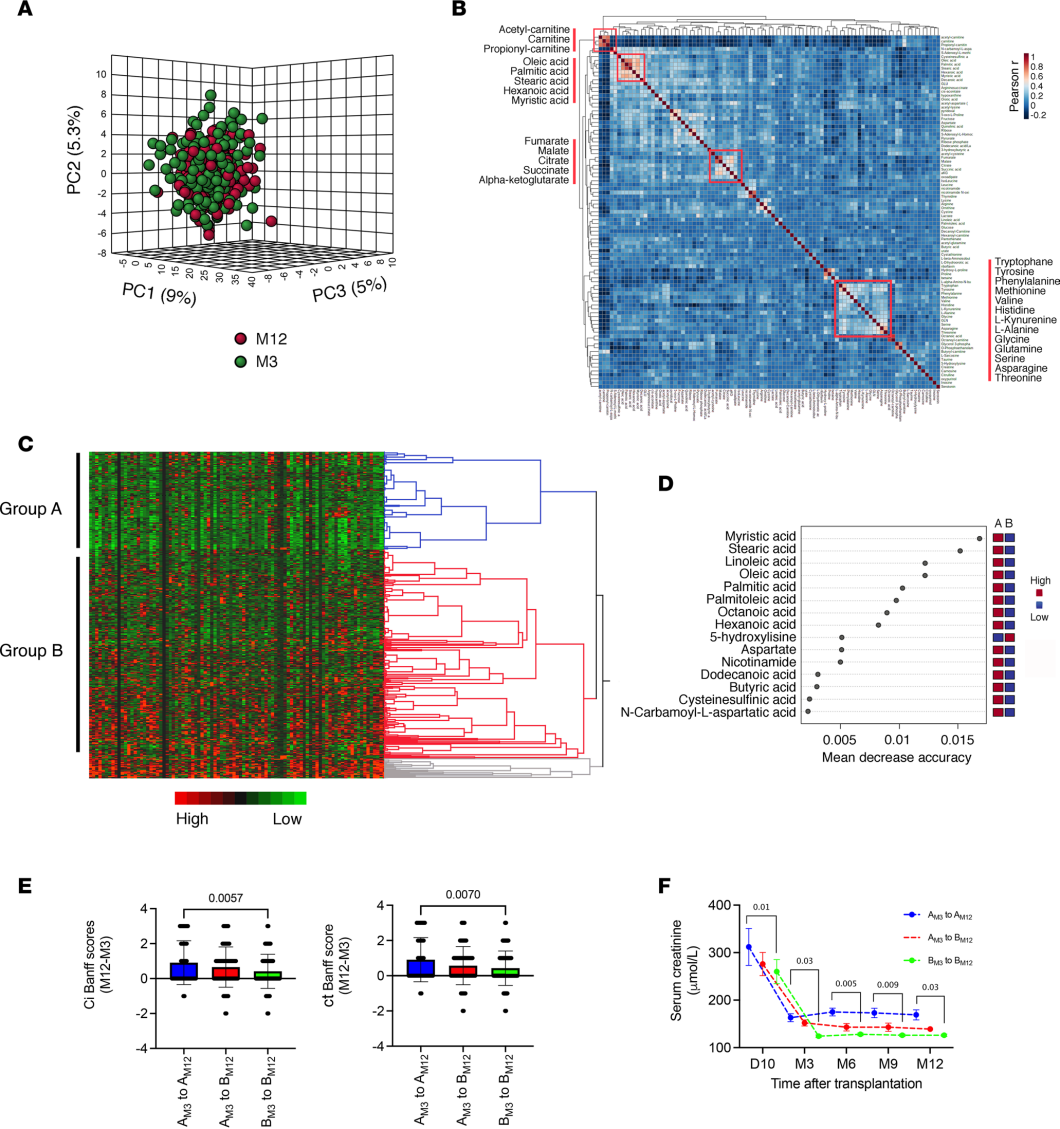
Urinary metabolome of KTRs after 3 and 12 months is enriched with long chain FAs. (**A**) Dimension reduction by principal component analysis (PCA) of all the metabolites identified in the 498 KTR urine samples collected 3 and 12 months after kidney transplantation. The plot shows the 3D scores between the selected PCs that best explain the variance of metabolites. (**B**) Overall correlation heatmap between metabolites using Pearson’s *r* scores in the 498 KTR urine samples collected 3 and 12 months after kidney transplantation. (**C**) Heatmap showing hierarchical clustering using Ward’s algorithm for all the metabolites identified in 498 urine samples collected 3 and 12 months after kidney transplantation. Blue, red, and gray colors indicate the 3 distinct clusters of the largest size, including group A and B. (**D**) Significant metabolites identified by random forest classification between group A and B. Metabolite importance (top 15) is calculated by mean decrease in accuracy for classification between group A and group B. Number of trees = 500, out of bounds error rate = 0.152. Class error rate: group A = 0.28 and group B = 0.09. Features are ranked by the mean decrease in classification accuracy after permutation. Dark red indicates that a feature (a metabolite) is enriched in a group. (**E**) Distribution of variations in the difference in interstitial fibrosis (ci) and tubular atrophy (ct) Banff scores between month 12 and month 3 (M12 and M3), according to changes of groups identified by hierarchical clustering in **B** between M3 and M12. A_M3_ to A_M12_ group, *n* = 33; A_M3_ to B_M12_ group, *n* = 33; B_M3_ to B_M12_ group, *n* = 33. The box plots depict the minimum and maximum values (whiskers), the upper and lower quartiles, and the median. The length of the box represents the interquartile range. *P* values were computed with a 1-way ANOVA followed by a Dunnett’s multiple-comparison test. (**F**) Distribution of renal function (plasma creatinine) according to changes of groups identified by hierarchical clustering in **B** between M3 and M12. A_M3_ to A_M12_ group, *n* = 33; A_M3_ to B_M12_ group, *n* = 33; B_M3_ to B_M12_ group, *n* = 33. *P* values were computed with an ordinary 2-way ANOVA followed by a Dunnett’s multiple-comparison test.

Urine metabolite profiling allows us to capture molecules released into the tubular lumen, potentially reflecting kidney pathological processes (19–21). We performed a targeted metabolomic profiling of 498 urine samples from 272 kidney transplant recipients (KTRs) collected 3 and 12 months after transplantation. The clinical characteristics of the study population are presented in Supplemental Table 1; supplemental material available online with this article; https://doi.org/10.1172/jci.insight.161783DS1 Principal component analysis (PCA) of all the metabolites detected in urine did not differentiate month 3 and month 12 samples (Figure 1A), indicating that the batch effect between the 2 time points was negligible. The whole metabolite-level correlation analysis highlighted the clustered variability of molecules involved in amino acid handling, carnitine derivates, and tricarboxylic acid cycle intermediates for the larger clusters, but the strongest signal was obtained in a cluster of long chain FAs (LCFAs) (Figure 1B). FAs are bound to albumin in the circulation, and their presence in urine may reflect glomerular permeability. However, in this cohort, their intensity was not correlated with proteinuria, which remained below 0.5 g/L (Supplemental Figure 1). This suggests that the presence of urinary FAs in a subset of patients reflects a tubular alteration, in line with previous studies (22).

We performed a hierarchical clustering based on global metabolomic variables at 3 and 12 months after transplantation to identify a minimal number of clusters of large size. Two main groups of patients were identified, which were referred to as group A and group B (Figure 1C). These groups showed substantial differences in urinary saturated LCFAs, including stearic acid (C18:0), palmitic acid (C16:0), and myristic acid (C14:0), which were top-ranked with the highest predictive ability (Figure 1D). The 2 groups were characterized by a significant difference in kidney function 3 months after kidney transplantation (estimated glomerular filtration rate, eGFR, shown in Table 1: 48.1 ± 2.3 mL/min/1.73 m^2^ versus 59.6 ± 1.8 mL/min/1.73 m^2^ in groups A and B, respectively; *P* = 0.0001). Interestingly, nearly twice as many patients from group A had a delayed recovery of function and higher frequencies of expanded criteria donors, but both groups had similar cold ischemia times (Table 1). Importantly, body mass index, diabetes, and statin use were similar between the 2 groups. In a multivariable logistic regression analysis, delayed graft function episode was the only feature independently associated with the metabolomic classification of the groups (Supplemental Table 2).

Further reflecting relevance of this metabolic classification over time, patients who remained in group A 12 months after transplantation (“A_M3_ to A_M12_”) had lower renal function compared with those who remained in group B 3 months and 12 months after transplantation (“B_M3_ to B_M12_”). Despite similar IF/TA scores at 3 months between group A and B (Table 1), the progression of IF/TA was significantly higher in the A_M3_ to A_M12_ group compared with the B_M3_ to B_M12_ group (Figure 1E). Patients who transitioned from group A at 3 months to group B at 12 months (“A_M3_ to B_M12_” group) had an intermediate phenotype in terms of renal function and progression to IF/TA (Figure 1, E and F). Delayed graft function episodes occurred in 44%, 27%, and 16% of patients in the A_M3_ to A_M12_, A_M3_ to B_M12_, and B_M3_ to B_M12_ groups, respectively (*P* < 0.0001) (Supplemental Table 3).

These results indicate that higher levels of LCFA in the urine are associated with reduced renal function and progression to chronic allograft injury. This association might be related to a more severe kidney injury at transplantation.

### The Cpt2^–^Acsl4^+^Acsl5^+^Acsm5^–^ signature is a feature of the transition to chronic kidney injury.

**Figure 2:**
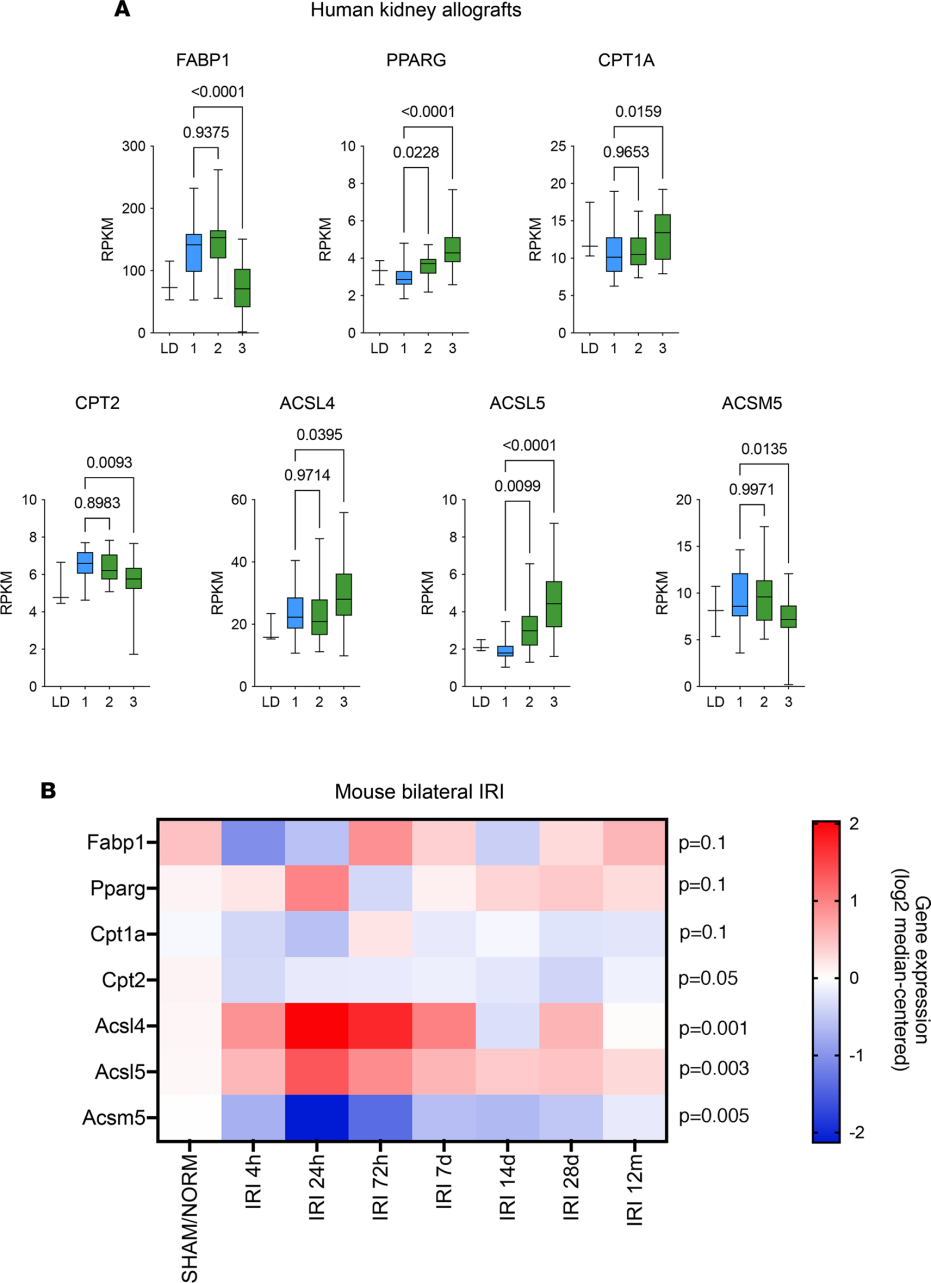
Dysregulation of FA metabolism is a feature of chronic allograft injury. (**A**) Expression of FABP1, PPARG, CPT1A, CPT2, ACSL4, ACSL5, and ACSM5 measured by RNA-Seq of mRNA isolated from kidney transplant biopsies in the group of 42 KTRs who recovered or progressed to fibrosis according to the computational model described (23), which identified 2 main transcriptional trajectories leading to kidney recovery or to sustained injury with associated fibrosis and renal dysfunction. LD, living donor (normal tissue); 1, successful repair state; 2, transition state; 3, chronically injured state; RPKM, reads per kilobase million. *P* values were computed in comparison with 1 using 1-way ANOVA followed by a Dunnett’s multiple-comparison test. (**B**) Expression of Fabp1, Pparg, Cpt1a, Cpt2, Acsl4, Acsl5, and Acsm5 transcripts measured by RNA-Seq of mRNA isolated from whole-mouse kidneys examined at different time points following bilateral ischemia/reperfusion injury (IRI, 3 to 4 mice per condition). *P* value was computed with a 1-way ANOVA. SHAM/NORM, normal kidney.

To identify components of FA metabolism determining recovery from initial IRI or progression to chronic kidney allograft injury, we analyzed the RNA-Seq transcriptional profiling of protocol kidney allograft biopsies of 42 KTRs at 3 and 12 months after kidney transplantation (23). As previously reported, the integrated analysis of the RNA-Seq data set delineated 2 distinct trajectories in response to AKI: successful repair or disease progression with a transition state to chronic kidney injury. A focused analysis on key genes in FA metabolism revealed that fatty acid binding protein 1 (FABP1), carnitine palmitoyltransferase 2 (CPT2), and acyl-CoA synthetase medium chain family member 5 (ACSM5) expression was progressively reduced along the transition to CKD, whereas the expression of peroxisome proliferator–activated receptor gamma (PPARG), carnitine palmitoyltransferase 1A (CPT1A), and acyl-CoA synthetase long chain family member 4 and 5 (ACSL4 and ACSL5) increased along the same trajectory (Figure 2A and Supplemental Figure 2).

To validate and generalize these findings, we examined RNA-Seq data obtained from renal tissue in mice up to 1 year after AKI after prolonged (21 minutes) bilateral IRI (18), which models the transition from acute to chronic injury. The time course analysis showed an early and sustained increase in Acsl4 and Acsl5 expression and a reduction of Cpt2 and Acsm5 expression (hereafter referred to as “Cpt2^–^Acsl4^+^Acsl5^+^Acsm5^–^” signature), similar to that observed in KTRs (Figure 2B), whereas the similitudes in the expression profiles of FABP1, PPARG, and CPT1A between the human data set and the IRI model were less obvious. This discrepancy can be explained by the fact that the injuries to which the kidney allograft is exposed are much more complex than in a simple model of IRI in the mouse. The result in terms of expression in the kidney allograft is the integration of all these stresses (alloimmune, ischemic, toxic, etc.) and can account for the differences observed between species.

**Figure 3:**
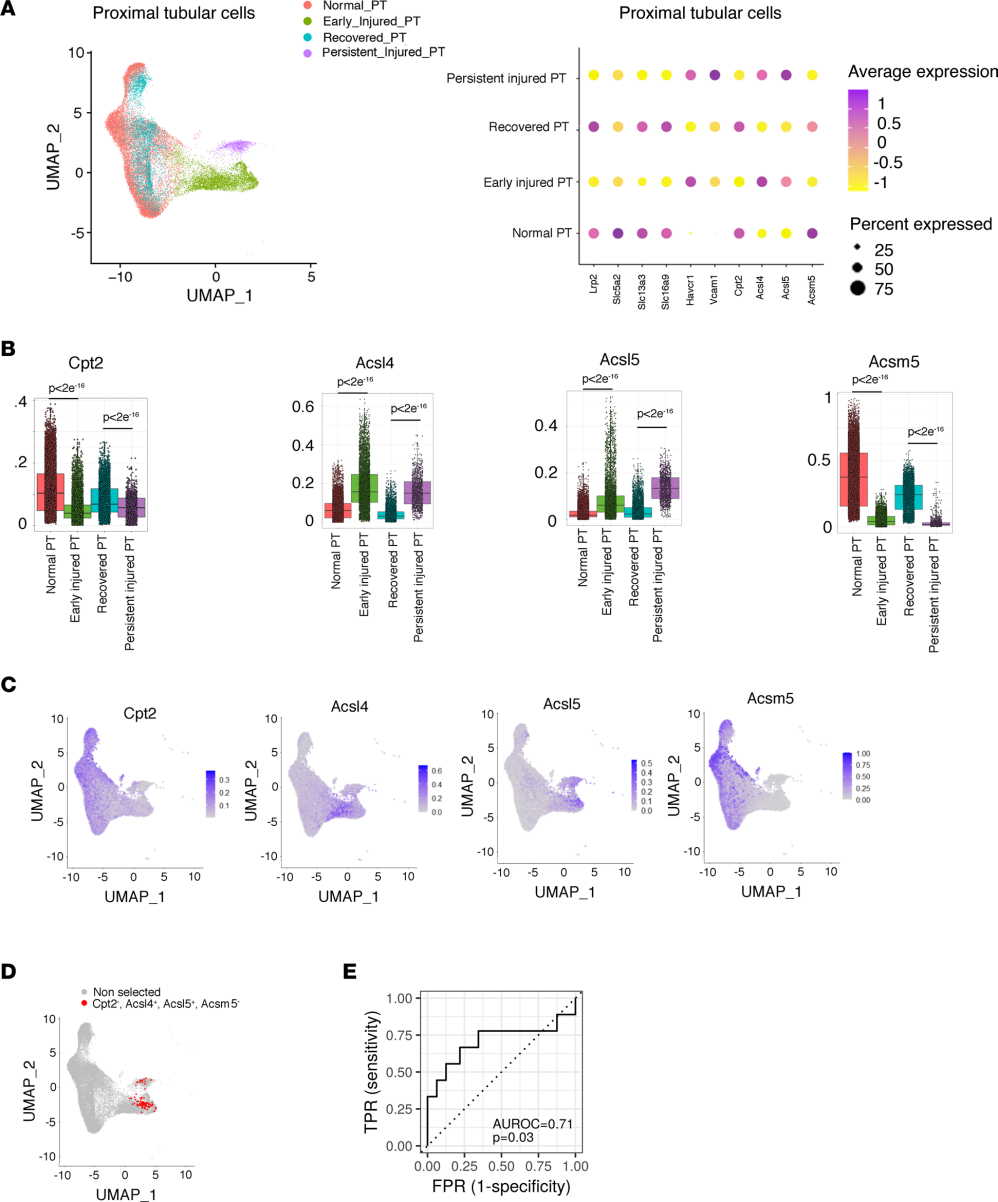
Dysregulation of FA metabolism persists in PTCs not recovering after AKI. (**A**) Uniform manifold approximation and projection (UMAP) (left panel) and dot plot (right panel) representation of the PTCs (*n* = 27,148 nuclei) selected from pooled mouse IRI and control kidney samples analyzed at different time points by snRNA-Seq. PTCs are depicted according to the definition of cell states based on the relative expression of differentiation markers (Lrp2, Slc5a2, Slc13a3, Slc16a9) and stress markers (Havcr1 and Vcam1) and the time points: normal PT (positive for Lrp2, Slc5A2, Slc13a3, Slc16a9 before injury), early injured PT (Havcr1 positive at 4 hours, 12 hours, 48 hours after IRI), recovered PT (positive for Lrp2, Slc5A2, Slc13a3, Slc16a9 at 14 days and 6 weeks), and persistent injured PT (positive for Havcr1, Vcam1 at 14 days and 6 weeks). (**B**) Expression levels of Cpt2, Acsl4, Acsl5, and Acsm5 in normal PT, early injured PT, recovered PT, and persistent injured PT. Box plots depict gene expression after application of Markov Affinity-based Graph Imputation of Cells (MAGIC). The box plots depict the minimum and maximum values (whiskers), the upper and lower quartiles, and the median. The length of the box represents the interquartile range. Pairwise comparisons using Wilcoxon rank sum test with continuity correction exhibit significant differences in gene expression among the different PTC clusters. (**C**) FeaturePlot representation of the expression of Cpt2, Acsl4, Acsl5, and Acsm5 in PTCs (*n* = 27,148 nuclei) selected from pooled mouse IRI and control kidney samples analyzed at different time points by snRNA-Seq. (**D**) FeaturePlot highlights in red (*n* = 92 dots) the PTCs with normalized gene expression of Acsl4 > 0.2 and Cpt2 < 0.05 and Acsl5 > 0.2 and Acsm5 < 0.05 (full house signature). (**E**) Receiver operating characteristic curve, and its underlying area (AUROC), for a model (logistic regression) predicting the binary ci score (ci ≤ 1 vs. ci > 1) 12 months after transplantation, using the expression levels of ACSL4, ACSL5, ACSM5, and CPT2 in kidney allografts 3 months after transplantation. *n* = 41. *P* value has been calculated with a Mann-Whitney *U* test. TPR, true positive rate; FPR, false positive rate.

The mouse bilateral IRI model was instrumental to better characterize this process at the cellular level. Single-nucleus RNA-Seq (snRNA-Seq) data from mouse kidney obtained from the first hours up to 6 weeks after IRI were integrated to study the PTC compartment after AKI. In the first hours/days after IRI, the PTCs displayed a substantial change in the transcriptional profile, with the expression of tubular cell injury markers (Havcr1 and Vcam1) and the reduction of PTC differentiation genes (e.g., Lrp2), leading to the characterization of potentially novel PTC clusters (PT0 to 3), which are referred to as “early injured PTCs” (Figure 3A and Supplemental Figure 3). Over time, the majority of the PTCs regained their normal transcriptional profile (hereafter called “recovered PTCs”), but we observed the persistence of a cluster of altered PTCs displaying markers of sustained injury (hereafter called “persistent injured PTCs”) (24). Strikingly, PTCs in the early and persistent damaged states displayed low levels of Cpt2 and Acsm5, and high levels of Acsl4 and Acsl5, compared with normal and recovered PTCs (Figure 3B). Furthermore, cells with the “full house” Cpt2^–^Acsl4^+^Acsl5^+^Acsm5^–^ signature were exclusively present in the early injured and persistent injured PTC clusters (Figure 3, C and D). These results indicate that the signature of dysregulated FA-related genes is engaged at the time of IRI and persists after the initial insult in a subset of PTCs.

Critically, in a multivariate analysis based on the data set of protocol kidney allograft biopsies with transcriptional profiling (23), the expression values of the 4-gene full house signature at month 3 were predictive of the progression of interstitial fibrosis (ci > 1) 12 months after transplantation (Figure 3E).

### The Cpt2^–^Acsl4^+^Acsl5^+^Acsm5^–^ signature is a feature of established CKD.

**Figure 4:**
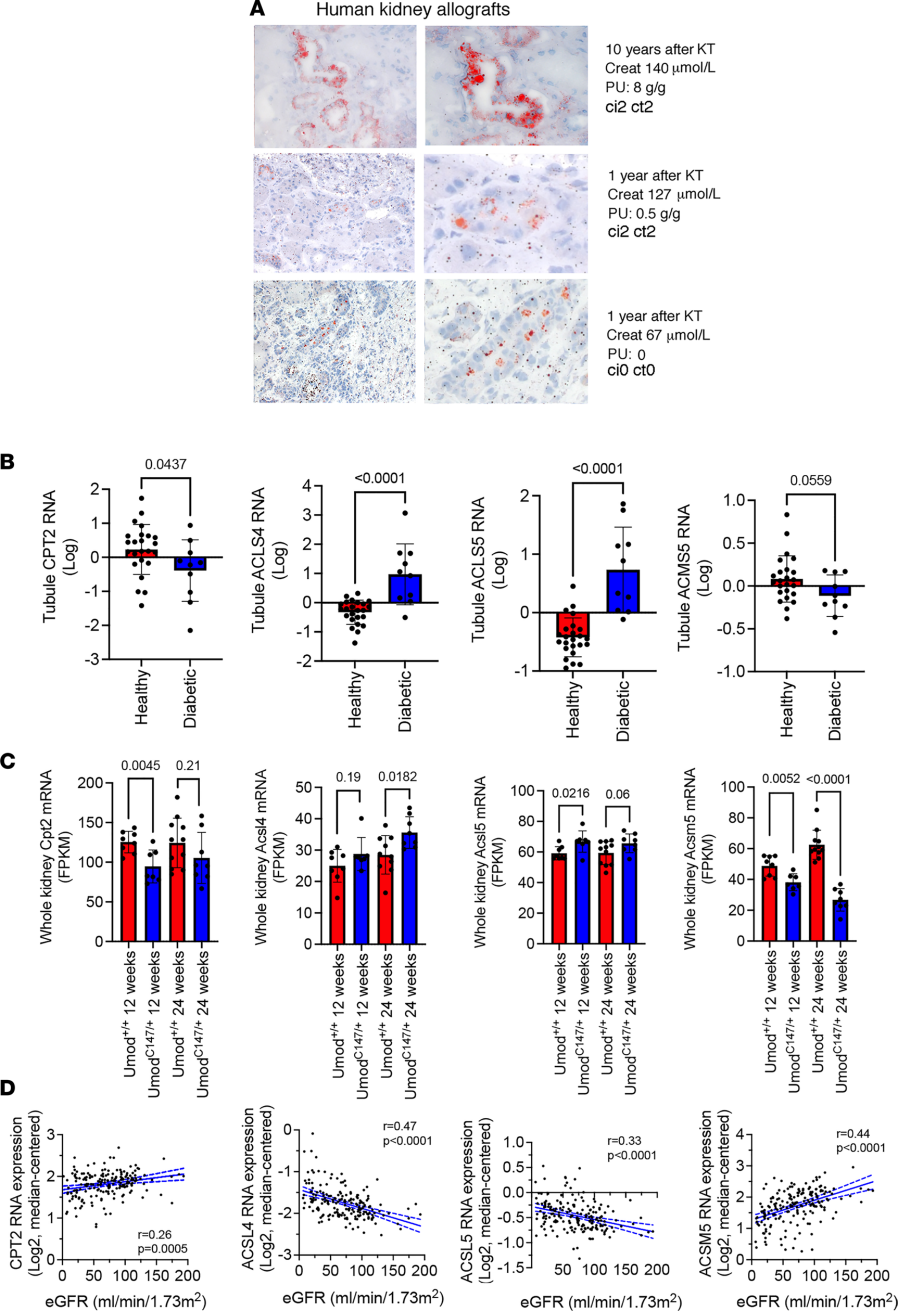
The Cpt2^–^Acsl4^+^Acsl5^+^Acsm5^–^ signature is a feature of established CKD. (**A**) Representative photomicrograph of neutral lipid accumulation in PTCs evaluated by Oil Red O staining in kidney allografts from 3 KTRs with chronic injury of various severity. Original magnification, ×40. Creat, creatinine; PU, urine protein-to-creatinine ratio. (**B**) Expression of Cpt2, Acsl4, Acsl5, and Acsm5 transcripts by expression profiling by array in the tubulointerstitial compartment of 24 healthy kidneys donors and 10 kidneys with diabetic kidney disease. Data are from public repositories (NCBI GEO accession GSE30122). Bars represent mean ± SD. *P* values were computed with a Student’s *t* test. (**C**) Expression of Cpt2, Acsl4, Acsl5, and Acsm5 transcripts by RNA-Seq in whole kidneys of 12- and 24-week-old UmodC147W/+ mice and wild-type mice (5 to 10 mice per condition). Data are from public repositories (GEO accession GSE102566). Bars represent mean ± SD. *P* values were computed with a Student’s *t* test. FPKM, fold-change per kilobase million. (**D**) Intrarenal expression of CPT2, ACSL4, ACSL5, and ACSM5 transcript levels as a function of the renal function (eGFR) of 201 healthy kidney donors and individuals with CKD. Data are from public repositories (Nephroseq). *R* is the Pearson’s correlation coefficient, and the *P* values were computed with a Student’s *t* test.

The presence of FA in urine may result from failure of esterification or breakdown of triacylglycerols (triglycerides) within PTCs (25). In turn, accumulation of triglycerides in PTCs is usually accompanied by excess FA in cells that promotes lipotoxicity (25–28). In line with this notion, we observed that PTCs of chronically injured kidney allografts accumulated lipids in the form of cytoplasmic lipid droplets evidenced by Oil Red O staining (which stains neutral lipids, such as triglycerides) (Figure 4A). However, it is difficult to differentiate an accumulation induced by the absorption of lipids bound to albumin (29, 30) in a context of advanced, and sometimes proteinuric, kidney disease, from a primary dysfunction of FA metabolism involved in kidney injury. Nevertheless, we observed that lipids also accumulated (albeit to a lesser extent) in PTCs from kidney allografts with minimal or no proteinuria (Figure 4A), suggesting that lipid metabolism may be impaired in a cell-intrinsic manner independent of reabsorptive activities in kidney allografts devoid of obvious structural lesions.

Having shown that FA metabolism was impaired in a population of injured PTCs unable to overcome cell stress initiated by IRI during progression to CKD, we wondered whether the Cpt2^–^Acsl4^+^Acsl5^+^Acsm5^–^ gene signature was a feature of established CKD.

We examined public repositories pertaining to the transcriptome of mRNA isolated from tubules of diabetic mice and whole-kidney tissue from a transgenic mouse model expressing mutant uromodulin (UmodC147W/+) (NCBI Gene Expression Omnibus [GEO] database accession no. GSE30122 and GSE102566, respectively) and found that expression of this set of FA-related genes had similar trends to that observed in the progression after IRI in the 2 CKD models: Cpt2 and Acsm5 were significantly downregulated in CKD, whereas Acsl4 and Acsl5 were upregulated (Figure 4, B and C).

Similar to our investigation in mice, we interrogated on Nephroseq (https://www.nephroseq.org/) a previously published expression array of the tubulointerstitial compartment from healthy donor nephrectomies and biopsies of patients with CKD of various etiologies and made gene expression correlation analyses (31) (Figure 4D). Correlation analysis of eGFR and CPT2, ACSL4, ACSL5, and ACSM5 expression levels in 201 healthy and CKD samples demonstrated that eGFR significantly negatively correlated with both ACSL4 and ACSL5 expression levels (*P* < 0.001) and positively correlated with CPT2 and ACSM5 (*P* < 0.0005). These results are consistent with the observations made above that ACSL4 and -5 were higher, and CPT2 and ACSM5 were lower, in injured kidneys. Together, these data indicate that the Cpt2^–^Acsl4^+^Acsl5^+^Acsm5^–^ signature is a feature of established CKD and could even be triggered independently of an ischemic episode.

### CPT2 inhibition induces epithelial phenotypic changes.

CPTs are rate-limiting enzymes that catalyze the transfer of LCFAs from cytoplasm into mitochondria, where FAO takes place, and a drastic reduction in FAO in the tubulointerstitial compartment generates energy failure, contributing to kidney fibrosis (10, 15). CPTs are present in the mitochondria in 2 isoforms, CPT1 and CPT2. Whereas the role of tubular CPT1A (which encodes a protein located at the outer mitochondrial membrane) in cellular and molecular changes associated with kidney fibrosis has been elucidated (15), the role of CPT2, which is located in the inner membrane, in kidney diseases remains unclear. A large whole-exome association analysis of 3,150 adults with CKD recently identified dominant signals in CPT2 gene (32), suggesting that its dysfunction may be involved in the pathogenesis of CKD.

**Figure 5:**
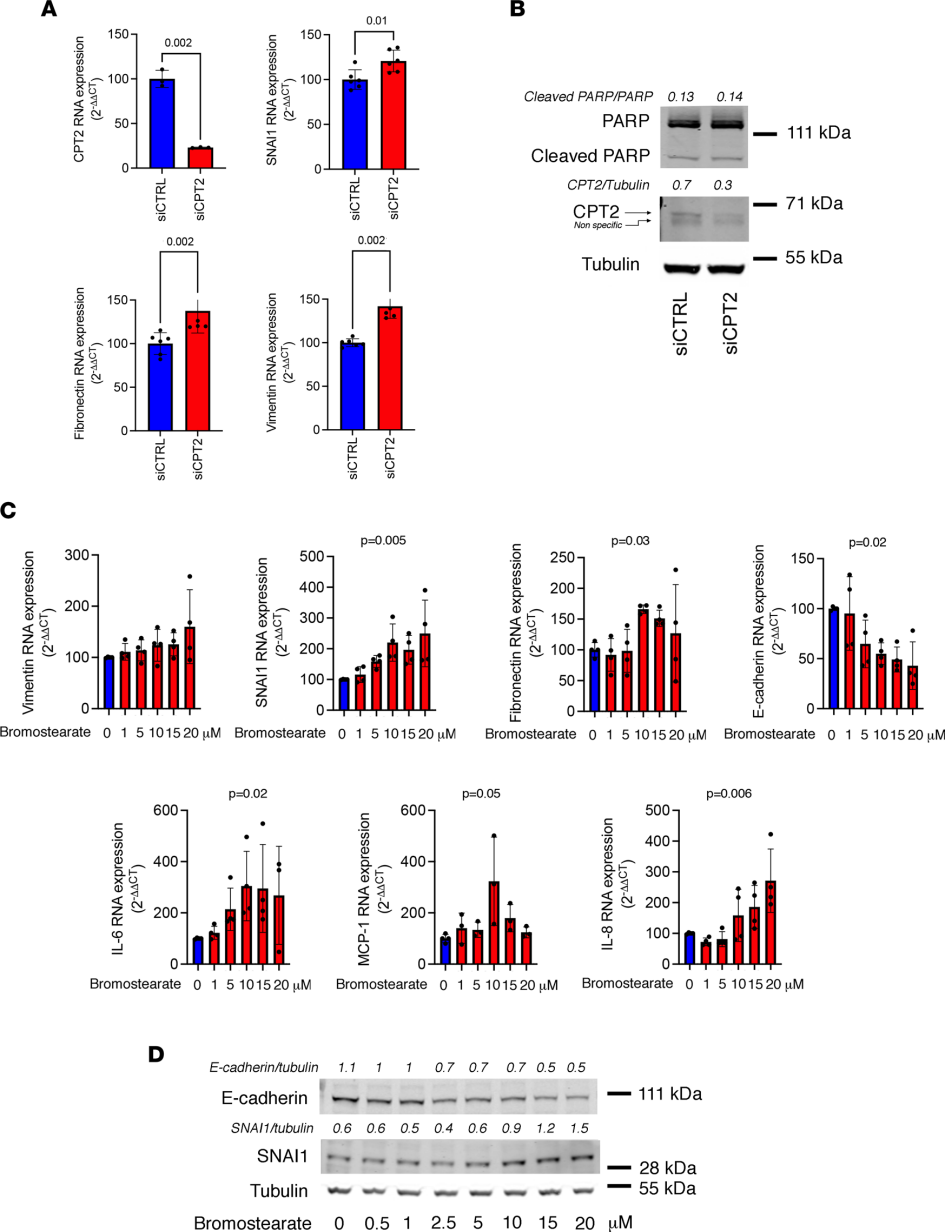
CPT2 inhibition drives epithelial phenotypic changes. (**A**) Relative expression of CPT2, SNAI1, fibronectin, and vimentin transcripts measured by real-time quantitative PCR (RT-qPCR) in HK2 cells transfected with CPT2 siRNA (siCPT2) or with control siRNA (siCTRL) for 48 hours. Bars represent mean ± SD. *P* values were calculated with a Student’s *t* test (4–5 replicates per condition). (**B**) Immunoblot representing the expression of PARP, CPT2, and tubulin in HK2 cells transfected with CPT2 siRNA (siCPT2) or with control siRNA (siCTRL) for 48 hours. The immunoblot shown is representative of 3 independent experiments. (**C**) Relative expression of vimentin, SNAI1, fibronectin, E-cadherin, IL-6, monocyte chemoattractant protein–1, and IL-8 transcripts measured by RT-qPCR in HK2 cells incubated with increasing concentrations of 2-bromostearate (2-bromo-octadecanoic acid) or DMSO for 24 hours (3–4 replicates per condition). Bars represent mean ± SD. *P* values were calculated with a 1-way ANOVA. (**D**) Immunoblot representing E-cadherin, SNAI1, and tubulin expression in HK2 cells incubated with increasing concentrations of 2-bromostearate (2-bromo-octadecanoic acid) or DMSO for 24 hours. The immunoblot shown is representative of 3 independent experiments.

We modeled the impact of CPT2 impairment in PTC homeostasis by performing in vitro studies in human kidney 2 (HK2) cells, a proximal tubule–derived cell line expressing CPT2 (Supplemental Figure 4) (33). HK2 cells in which CPT2 is repressed by siRNA-mediated RNA interference activate a transcriptional program reminiscent of partial epithelial-mesenchymal transition, for instance upregulation of the transcriptional regulator snail family transcriptional repressor 1 (SNAI1; a master regulator of biological processes responsible for renal fibrogenesis; refs. 4, 5), vimentin, and fibronectin (Figure 5A). The inhibition of CPT2 did not increase apoptotic activity [monitored by cleavage of poly(ADP-ribose) polymerase, PARP] (Figure 5B). In line with this, disruption of FAO in HK2 cells by 2-bromostearate (34, 35) (Supplemental Figure 6A) engaged a similar molecular phenotype (Figure 5, C and D), albeit with the expression of inflammatory markers not present in CPT2-invalidated cells. For example, concentrations of bromostearate more than 5 μM induced a level of expression of cytokines such as IL-6 or IL-8 at least 3 to 4 times the expression levels of control conditions. This indicates that when CPT2 expression is repressed, PTCs undergo epithelial phenotypic changes associated with development of progressive fibrotic kidney disease (4, 5).

### ER stress represses CPT2 expression.

**Figure 6:**
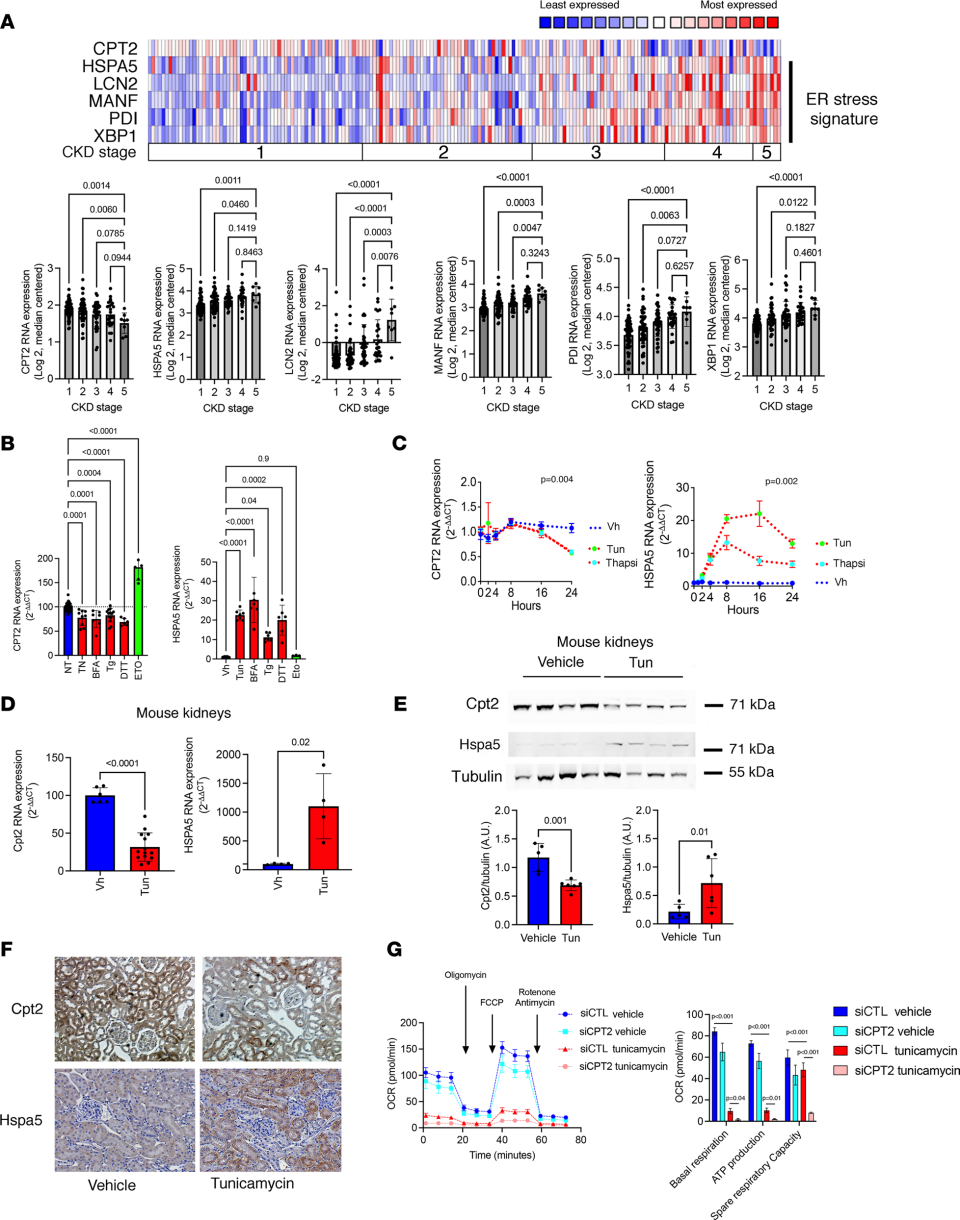
ER stress reduces CPT2 expression. (**A**) Correlation between CKD stages and ER stress genes’ expression from the tubulointerstitial compartment of 201 individuals with CKD. Colors are *z* score–normalized to depict relative values within rows. *P* values were calculated with a 1-way ANOVA followed by a Dunnett’s multiple-comparison test. (**B**) Expression of HSPA5 and CPT2 measured by RT-qPCR in HK2 cells incubated with 2.5 μg/mL tunicamycin (Tun), 5 μg/mL brefeldin A (BFA), 0.25 μM thapsigargin (Tg), 1 μM dithiothreitol (DTT), 100 μM etoposide (Eto), or DMSO for 24 hours (*n* = 7–8). Bars represent mean ± SD. *P* values were calculated with a 1-way ANOVA followed by a Dunnett’s multiple-comparison test. (**C**) Time course analysis of the relative expression of CPT2 and HSPA5 transcripts measured by RT-qPCR in HK2 cells incubated with either with vehicle or with 0.25 μM Tg, 5 μg/mL BFA, or 2.5 μg/mL Tun for up to 24 hours (4 replicates). Bars represent mean ± SD. *P* values were computed with 2-way ANOVA. (**D**) Expression of Cpt2 and Hspa5 transcripts by quantitative PCR in kidney cortex of mice 48 hours after intraperitoneal injection of 1 mg/kg Tun or DMSO (*n* = 5 in DMSO group and 14 in Tun group). Bars represent mean ± SD. *P* values were computed with a Student’s *t* test. (**E**) Immunoblot representing Cpt2, Hspa5, and tubulin protein expression in kidney cortex of mice 48 hours after intraperitoneal injection of 1 mg/kg Tun or DMSO (*n* = 4). Bars represent mean ± SD. *P* values were computed with a Student’s *t* test. (**F**) Representative photomicrograph of Cpt2 and Hspa5 expression in kidney cortex of mice 48 hours after intraperitoneal injection of 1 mg/kg Tun or DMSO (*n* = 5). Original magnification, ×40. (**G**) OCR measured by Seahorse bioanalyzer in HK2 cells 48 hours posttransduction with siRNA targeting CPT2 under basal conditions and in response to incubation with DMSO or 2.5 μg/mL Tun for 24 hours. *P* values were computed with a Student’s *t* test, *n* = 5–6.

To investigate the potential mechanisms determining the reduction of CPT2 expression in the kidney, we focused on ER stress, which is known to be activated in the AKI response (36–38) and drives tissue remodeling associated with CKD (39–41). Moreover, ER stress profoundly reshapes the cell lipidome (42). Supporting a role of ER stress in regulating CPT2 expression in the kidney in the course of CKD, we observed in the cohort of 201 CKD patients and healthy donors cited above (31) that ER stress response genes (PDI, heat shock protein family A member 5 [HSPA5], and XBP1) (43), and biomarkers of ER stress–related kidney diseases (LNC2 and MANF) (39, 44), were highly expressed in the tubulointerstitial compartment of patients with low eGFR, and, importantly, CPT2 expression was reduced in kidneys expressing the ER stress signature (Figure 6A). ER stress induced by tunicamycin (Tun), an inhibitor of glycosylation, brefeldin A (BFA, a lactone that inhibits protein transport from the ER to the Golgi complex indirectly by preventing association of COP-I coat protein 1), thapsigargin (Tg, an inhibitor of SERCA), and dithiothreitol (DTT, a reducing agent) activated the unfolded protein response (UPR) and repressed CPT2 levels in HK2 cells (Figure 6B and Supplemental Figure 5). Etoposide, a topoisomerase inhibitor which served as a control to promote apoptosis, did not induce ER stress and even increased CPT2 expression, indicating that CPT2 downregulation was not a nonspecific consequence of ongoing cell damage. Time course analysis of CPT2 transcript levels in HK2 cells after ER stress activation showed CPT2 downregulation starting between 8 hours and 16 hours after stimulation, indicating a late and likely indirect regulatory process (Figure 6C). A diminished expression of Cpt2 was also found in whole kidney extracts and proximal tubules of mice injected with Tun and that developed renal ER stress (Figure 6, D–F).

We next examined the metabolic profile of mitochondria of ER-stressed PTCs using Seahorse Bioanalyzer. We first measured the oxygen consumption rate (OCR) of HK2 cells (Supplemental Figure 6A) and found that OCR was higher when we added stearate (an FAO substrate) to cells, indicating that these cells efficiently metabolized stearate. We also found that FAO was the key contributor to intracellular ATP production in HK2 cells, as bromostearate (an FAO inhibitor) markedly reduced intracellular ATP production capacity in these cells, and increased extracellular acidification rate, consistent with a switch from FAO to anaerobic glycolysis. In ER-stressed cells, the metabolic profiling of mitochondria resulted in inhibition of basal and spare mitochondrial respiration as well as decreased ATP production capacity (Supplemental Figure 6B). Thus, activation of the ER stress response in PTCs resulted in an energetic depression consistent with a defect in FA degradation. Critically, the depressant effect of ER stress on mitochondrial energy metabolism was multiplied by the invalidation of CPT2 by RNA interference (Figure 6G), which underlines that ER stress did indeed affect the activity of CPT2.

### ER stress and CPT2 inhibition engage an auto-amplification loop leading to lipotoxicity.

**Figure 7:**
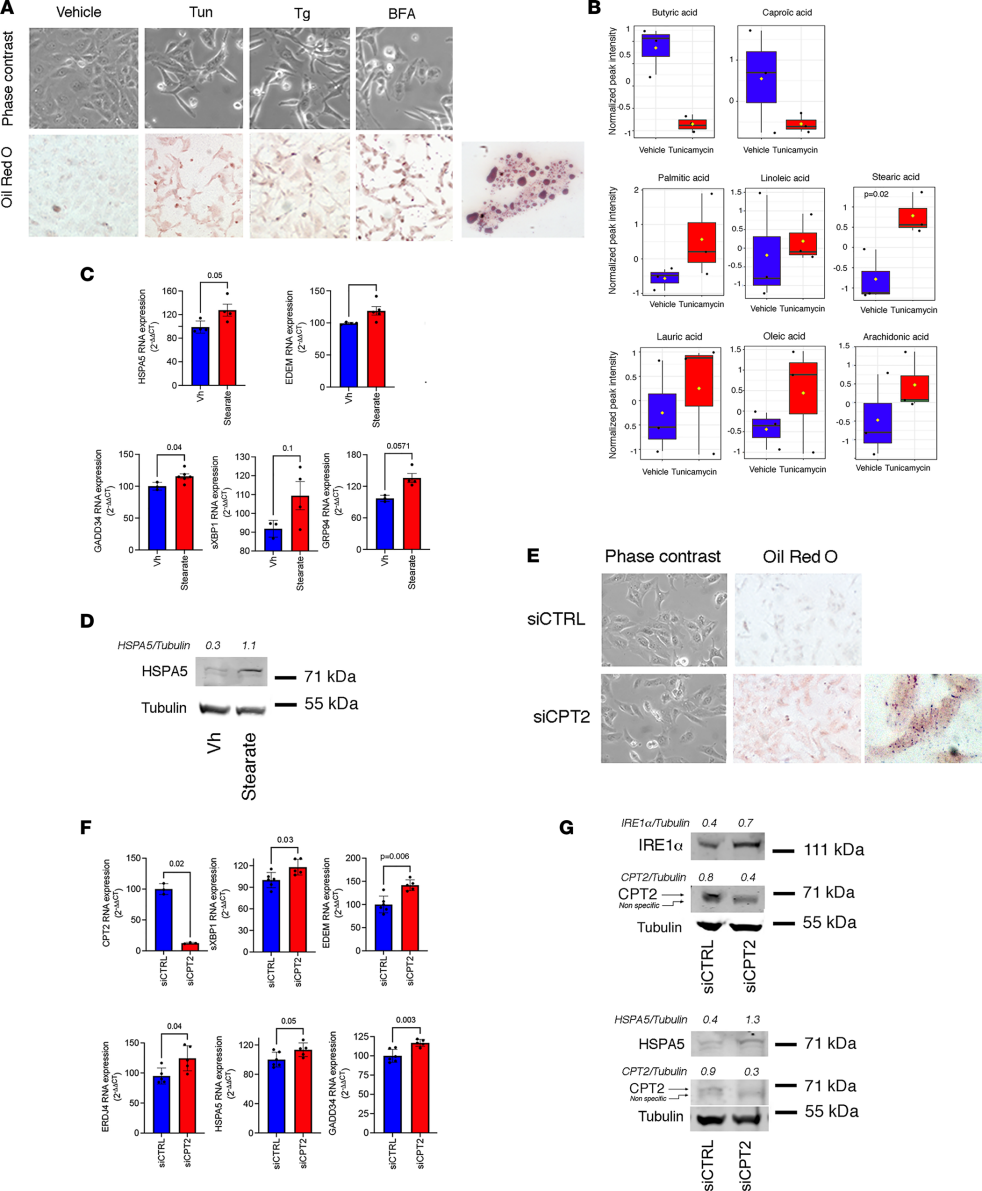
ER stress and CPT2 inhibition engage an auto-amplification loop leading to lipotoxicity. (**A**) Representative photomicrograph of phase contrast and Oil Red O staining of HK2 cells incubated either with DMSO or with 2.5 μg/mL Tun, 0.25 μM Tg, or 5 μg/mL BFA for 24 hours (3–4 replicates per condition). Original magnification, ×10. (**B**) Distribution of various medium chain FA and LCFA standardized levels measured by mass spectrometry in HK2 cells incubated with 2.5 μg/mL Tun for 24 hours (3 replicates). (**C**) Relative expression of HSPA5, EDEM, GADD34, spliced XBP1 (sXBP1), and GRP94 measured by RT-qPCR in HK2 cells incubated with 500 μM of stearic acid for 24 hours (3 replicates per condition). Bars represent mean ± SD. *P* values were calculated with Student’s *t* test. Vh, vehicle. (**D**) Immunoblot representing HSPA5 and tubulin protein expression in HK2 cells incubated with 500 mM of stearic acid for 24 hours (3 replicates per condition). (**E**) Representative photomicrograph of Oil Red O staining of HK2 cells transfected with siCPT2 or with siCTRL for 48 hours. Original magnification, ×10. (**F**) Relative expression of CPT2, spliced XBP1 (sXBP1), EDEM, ERDJ4, HSPA5, and GADD34 measured by RT-qPCR in HK2 cells transfected with siCPT2 or with siCTRL for 48 hours (5–6 replicates per condition). Bars represent mean ± SD. *P* values were calculated with Student’s *t* test. (**G**) Immunoblots representing IRE1α, HSPA5, CPT2, and tubulin protein expression in HK2 cells transfected with siCPT2 or with siCTRL for 48 hours.

Accumulation of lipid intermediates in nonadipose cells has a detrimental effect on cell functions and, in particular, ER membrane perturbations caused by changes in lipid accumulation and saturation that can activate sensors of the UPR (45, 46). To test this possibility in PTCs, we induced ER stress with Tun, and we performed metabolomics analysis in the HK2 cell lysates after 24 hours of incubation. ER stress profoundly reshaped the intracellular metabolic profile of HK2 cells (Supplemental Figure 7). ER stress led to intracellular lipid accumulation in HK2 cells (Figure 7A) and, more precisely, was associated with a relative increase in LCFA compared with the control condition (DMSO), whereas short chain FAs were downregulated (Figure 7B), suggesting that LCFAs accumulate in ER-stressed PTCs. In agreement with defective FA degradation in mitochondria, lipidomic data indicated that LCFAs accumulated in cells incubated with ER stressors. LCFA-CoA are metabolite intermediates involved in phospholipid (PL) synthesis but also form triacylglycerol (triglycerides) and cholesterol ester (CE-Chol) as a storage pathway. We found that Tun drastically increased the relative amounts of triglycerides and CE-Chol, with a specific enrichment in LCFAs (Supplemental Figure 8). Moreover, we found a dramatic decrease in the amount of lysophospholipids (LysoPLs). LysoPLs are PLs in which an FA has been removed, such that this FA can be used for other cues such as FAO. This suggests that the need of FAs with PLs as a source for FAO was reduced. These results are consistent with a defect in FAO and cellular accumulation of FAs, with 2 consequences: an increase in triglycerides and CE-Chol and a decrease in LysoPL amounts.

Interestingly, this metabolic profile shared qualitative similarities with the urine lipid profile we observed in KTRs (Figure 1D), and stearic acid, a saturated LCFA with lipotoxic properties (47), was the most strongly upregulated FA in ER-stressed cells and a top metabolite discriminating KTRs that belonged to the group of KTRs with evolutive chronic allograft injury. LCFAs are potent ER stress inducers (48, 49), and we observed that HK2 cells incubated with stearate or palmitate activated the UPR (Figure 7, C and D, and Supplemental Figure 9). Critically, HK2 cells in which CPT2 was repressed by RNA interference accumulated intracellular lipids (Figure 7E), and expressed markers of ER stress, for instance markers of the activity of the IRE1/XBP1 pathway (sXBP1, EDEM, and ERJ4), the ATF6 pathway (HSPA5), and the PERK pathway (GADD34) (Figure 7, F and G). This observation raises the possibility that an amplification loop can occur after ER stress has driven CT2 repression, which participates in lipid accumulation and lipotoxicity, and in turn, promotes ER stress.

## Discussion

Chronic injury ultimately leads to a decline in kidney graft function and failure. Although the most recent studies have focused on immune-mediated injury, there is a critical need to identify mechanisms of injury and to understand the mechanisms of late kidney allograft graft failure. By identifying potentially novel metabolomic signatures and gene candidates, we provide directions for future studies of chronic kidney allograft injury biochemical pathophysiology and potential therapeutic targets. We provide evidence that a dysregulation of FA metabolism in PTCs is likely a driver for the progression to IF/TA in kidney allografts, mediated at least in part by lipotoxicity. This progression is operated by a set of PTCs imprinted immediately by IRI with a metabolic signature that persists months after IRI and that accompanies the progression to chronic lesions leading to IF/TA. The persistence of these cellular disturbances, the inability to resolve cell stress, and the proportion of tubules presenting these abnormalities compared with healthy tubules appeared critical in the progression of organ dysfunction. Here, we demonstrate that the ER stress response participated in FA metabolism dysregulation in the kidney and engaged an amplification loop based on CPT2 downregulation and lipotoxicity, which in turn promoted ER stress. This sustained, deleterious process may explain why a subset of PTCs cannot restore cellular homeostasis after initial IRI. Critically, CPT2 is induced by PPARα agonists and, as such, is a validated therapeutic target (50). Finally, we supply strong evidence supporting the role of ACSL4 in the IRI–IF/TA transition and probably in CKD. ACSL4 plays a role in sensitization of cells to ferroptosis and is inhibited by thiazolidines independently of PPARα agonism (51, 52).

The general pathogenic mechanism of lipotoxicity in the kidney has been proposed to be an overload of intracellular free FAs, associated with an accumulated triglyceride pool, with subsequent reactive oxygen species production and inflammasome activation (53), ceramide accumulation (54), mitochondrial dysfunction (55), DNA damage (56), cell cycle arrest (57), enhanced lysosomal activity and impaired autophagy (55), and ER stress (58). All these perturbations occurring within PTCs drive tubulointerstitial inflammation, fibrosis with PTC apoptosis, and secretion of a combination of profibrogenic and proinflammatory factors (25). However, the mechanisms that govern intracellular lipid accumulation in the diseased kidneys remain undefined. The consensus model is based on glomerular damage, leading to an increased amount of FA-bound albumin filtered through glomeruli, forcing PTCs to reabsorb excessive quantities of FA-bound albumin, ultimately resulting in tubulointerstitial injury through the mechanism mentioned above (29). Our results indicate that intracellular lipid accumulation may occur without significant proteinuria (and albumin-bound FAs). We propose that cell-intrinsic perturbation of FA metabolism, associated with lipid accumulation, are triggered by IRI and persist in a subset of PTCs that survive in a persistent state of stress. In this model, primitive disturbances of the molecular circuitries that affect intracellular FA homeostasis, for instance CPT2 downregulation and activation of the metabolic pathways that produce LCFA PLs by ACSLs, may result in lipotoxicity. This does not exclude the possibility the excessive FA reabsorption also occurs in parallel during the progression to chronic injury. Mechanistically, our model is based on an auto-amplification loop involving ER stress and CPT2 downregulation, which drives lipid accumulation and lipotoxicity, resulting in ER stress. Multiple causes of ER stress coexist in the kidney upon IRI, CKD, and the transition between AKI and CKD (16, 39, 40, 44, 59–62). ER stress affects FA metabolism (63–65), and an overload of intracellular FAs induces ER stress (45, 46). We show here that this fundamental cell stress process engaged in the response to kidney injury is both a cause and a consequence of FA metabolism dysregulation and drives cellular injury and tissue remodeling through multiple ways, including CPT2 downregulation. However, the question of the precedency of ER stress or FA metabolism abnormalities activated after IRI remains to be evaluated. In addition, the molecular mechanisms by which the UPR represses CPT2 expression await clarification. The kinetics of CPT2 transcripts’ downregulation favors a late process, possibly involving intermediates, for example the transcription factor ATF6, which downregulates the CPT2 activator PPARα (63). It is interesting to note that an impairment in CPT2 activity can have harmful consequences on the progression of CKD besides its impact on PTC physiology. For example, it has been demonstrated in other cell types involved in renal fibrogenesis, such as endothelial cells and macrophages, that an invalidation of CPT2 accompanied by a decrease in the FAO oriented the phenotype of the cells toward epithelial phenotypic changes and a proinflammatory pathogenic profile (66, 67). Furthermore, neonatal CPT2 deficiency presents within days after birth and is characterized by structural malformations including cystic renal dysplasia (68), indicating that CPT2 is a critical enzyme in kidney physiology.

Our results demonstrate that urinary profile reflects ongoing lipotoxicity, with enrichment in FAs associated with altered renal function, such as saturated LCFAs with nephrotoxic properties (47). Most urinary metabolites are very hydrophilic, though clearly, trace amounts of lipids and FAs contribute a number of chemicals to the urinary metabolome. This is in striking contrast with the composition of serum, which is particularly rich in lipids (69). The mechanisms of production of these FAs in the urine (passive or active secretion), though they do not represent products of glomerular filtration, are unknown and remain to be determined. Many mechanisms may be involved, and the most obvious possibility is that LCFAs accumulate in PTCs in response to ER stress and are released and/or secreted in the urine. The regulated expression of transporters or efflux pumps at the apical membrane may play a role. A possible prognostic value of the presence of cytotoxic saturated LCFAs in urine remains to be established as well.

In summary, we identified a renal FA-related gene signature made of CPT2, ACSL4, ACSL5, and ACSM5, which is systematically associated with IRI, with transition to chronic injury, and with established CKD in mouse models and KTRs. CPT2 and ACSL4, which are critical regulators of FAO and ferroptosis, respectively, are known therapeutic targets. Additional studies should be undertaken to demonstrate the prognostic value of this gene signature. Mechanistically, we provide evidence that peri-transplantation ischemic damage initiates a profound reprogramming of FA metabolism in PTCs that persists long after IRI in a subset of these cells that cannot resolve cellular stress as they are engaged in an auto-amplification loop involving ER stress, CPT2 repression, and lipotoxicity. These cells may actively participate in chronic tissue remodeling through the acquisition of a fibrogenic phenotype.

## Methods

### Study populations

#### Necker KTR cohort for urinary metabolome.

All the consecutive patients (*n* = 405) who received a kidney transplant at our center from January 2010 to June 2012 were considered for this prospective, longitudinal, single-center cohort study. The reasons for exclusion were noninclusion criteria (*n* = 48), primary nonfunction/early graft loss (*n* = 12), other study with urine monitoring (*n* = 16), patients’ death within the first 6 months (*n* = 7), and early loss to follow-up (*n* = 22). Posttransplantation, urine was collected at months 3 and 12 for 272 out of 300 individuals initially included in the study. In total, 498 KTRs’ urinary metabolomes were analyzed: 248 samples were analyzed at month 3, and 250 samples were analyzed at month 12. Among them, 207 KTR samples were analyzed both at month 3 and at month 12. Urine samples were centrifuged at 1,000*g* for 10 minutes at 4°C within 4 hours of collection. The supernatant was collected after centrifugation and stored with protease inhibitors (Thermo Fisher Scientific) at –80°C. The urine samples used in this study have been part of a previously published analysis (70).

#### Leuven KTR cohort for kidney allograft RNA-Seq.

A total of 41 KTRs were enrolled at the University Hospitals of Leuven (23). In each case, protocol biopsies were performed at 4 time points: before implantation (pre, kidney flushed and stored in ice), after reperfusion (post, at the end of the surgical procedure), and at 3 and 12 months after transplantation. Genome-wide gene expression profiling using RNA-Seq was performed in kidney allograft recipients as previously described (23). Based on RNA-Seq profiling of protocol biopsies, a computational model to characterize the transition from AKI to CKD has been developed, as previously described (23). Briefly, a pseudotime analysis including all 3-month and 12-month samples was performed to cover this transition. The pseudotime line separated into 2 branches: a branch with transcriptomes depicting the progression to fibrosis over time (called “transition” and “progression”) and an opposite branch with transcriptomes moving toward recovery (called “recovery”).

### Targeted metabolomics

HK2 cells were washed twice with ice-cold PBS, drained, snap-frozen in liquid nitrogen, and stored at –80°C until analyses. Urine was centrifuged 5 minutes at 4,000*g* at 4°C, and the supernatant was stored at –80°C until analyses. After addition of an extraction solution made of 50% methanol, 30% acetonitrile, and 20% water (71) (1 mL/1.10^6^ cells or 500 μL for 20 μL urine), the samples were vortexed for 5 minutes at 4°C, then centrifuged at 16,000*g* for 15 minutes at 4°C. The supernatants were collected and separated by liquid chromatography-mass spectrometry (LC-MS) using SeQuant ZIC-pHilic column (MilliporeSigma). The aqueous mobile-phase solvent was 20 mM ammonium carbonate plus 0.1% ammonium hydroxide solution, and the organic mobile phase was acetonitrile. The metabolites were separated over a linear gradient from 80% organic to 80% aqueous for 15 minutes. The column temperature was 50°C, and the flow rate was 200 μL/min. The metabolites were detected across a mass range of 75 to 1,000 *m/z* using the Q Exactive Plus mass spectrometer (Thermo Fisher Scientific) at a resolution of 35,000 (at 200 *m/z*) with electrospray ionization and polarity switching mode. Lock masses were used to ensure mass accuracy below 5 ppm. The peak areas of different metabolites were determined using Thermo Fisher Scientific TraceFinder software using the exact mass of the singly charged ion and known retention time on the HPLC column.

Our metabolomics analyses are focused on small polar compounds in central carbon metabolism. We applied an established and referenced method for sample extraction and LC-MS analyses using pHILIC HPLC column for polar metabolites’ separation (71). Notably, we used the same extraction solution for cells and urine samples. As a part of the routine analytical pipeline, we applied the recommendations of the metabolomics Quality Assurance & Quality Control Consortium. The routine quality controls included regular equipment maintenance (Thermo Fisher Scientific) and the use of standard operating procedures for sample extraction, storage, and analyses. General practices also included weekly test runs to ensure system stability and quality of runs. Regarding the quality controls (QCs) in relation to this study, we used a) pooled interstudy QC, b) process and extraction blanks, c) system stability blanks, d) solvent blanks, e) long-term reference standard interlaboratory QC mix to ensure system stability, and f) samples that were blinded and loaded in randomized order. The analyses of pooled samples’ QC showed no significant difference in metabolites’ levels between QCs.

Data analysis was performed in the MetaboAnalyst 5.0 software (72). Each urine metabolite was normalized to urine creatinine level to avoid dilution bias. Samples were normalized by sum and each metabolite level was adjusted by autoscaling. PCA was performed including all the metabolites identified in KTR urine at 3 and 12 months to determine the impact of the time on urine metabolome. Hierarchical clustering was performed using the Ward method including all the metabolites identified in the KTR urine.

### Animal experiments

#### Tun injection.

At 12 weeks old, C57BL/6 background male mice (Charles River Laboratories) were intraperitoneally injected with Tun (MilliporeSigma, T7765) (1 mg/kg) or vehicle (DMSO) at day 0, and mice were sacrificed 2 days postinjection (*n* = 4–5 mice per condition). Total RNA was extracted from kidneys using the RNeasy Mini Kit (QIAGEN) according to the manufacturer’s protocol.

#### UmodC147W/+ mice.

A detailed description of the methods for the UmodC147W/+ mouse line of the C57BL/6J genetic background was previously reported (40). Public repositories pertaining to the transcriptome of mRNA isolated from whole-kidney tissue from mutant mice and littermate controls at multiple time points, including 12 and 24 weeks (GEO database accession GSE102566), were analyzed for Acsl4, Acsl5, Acsm5, and Cpt2 expression.

#### Bilateral IRI for bulk RNA-Seq.

The surgical procedure has been detailed (18). A 21-minute warm renal IRI was performed on 10- to 12-week-old (25–28 g) C57BL/6CN male mice. The procedure of bulk RNA-Seq of kidneys after bilateral IRI has been detailed (18). GEO database accession GSE98622 data were analyzed for Acsl4, Acsl5, Acsm5, and Cpt2 expression at 4 hours; 1, 3, 7, 14, and 28 days; and 12 months after IRI (*n* = 3–4 mice per condition).

#### Bilateral IRI for snRNA-Seq.

snRNA-Seq data were obtained from experimental IRI models previously described (18). Bilateral IRI was induced by clamping the renal pedicle with a nontraumatic microaneurysm clamp for 18.5 minutes in 8- to 10-week-old male C57BL/6J mice. Mice were euthanized at 4 hours, 12 hours, 48 hours, 14 days, and 6 weeks after IRI (73). In the other data set, bilateral IRI was induced by clamping the renal pedicle with a nontraumatic microaneurysm clamp for 15 minutes in 10- to 12-week-old, 25–28 g, male C57BL/6J mice. Mice were euthanized at 64 hours, 96 hours, and 28 days after IRI (74). The procedure of snRNA-Seq of kidneys after bilateral IRI has been detailed in (73, 74). GEO database data (GSE151167 and GSE139107) were analyzed for Acsl4, Acsl5, Acsm5, and Cpt2 expression.

### General strategy of snRNA-Seq data analysis

The experimental protocol is shown in Supplemental Figure 3; 2 data sets were analyzed (73–75). Seurat v3.2.0 in R v4 was used for analyses, including normalization, scaling, and clustering of nuclei. First, we analyzed each data set separately and excluded nuclei with fewer than 150 or more than 8,000 genes detected. We also excluded nuclei with a relatively high percentage of unique molecular identifiers mapped to mitochondrial genes (>1) and ribosomal genes (>1, for normal kidney sample, and >2, all other samples). We performed curated doublet removal based on known lineage-specific markers.

The samples from the 2 different data sets were integrated, and Seurat standard workflow splitting by data set was used to avoid batch effect. We extracted the PT clusters (normal, early injured, recovered, and persistent injured) based on the time point after IRI and the phenotype. Normal_PT (S1, S2, and S3) corresponds to time point 0 (16,509 nuclei); Early injured_PT (PT0, PT1, PT3) corresponds to time points 4 hours, 12 hours, and 48 hours (5,401 nuclei); Recovered_PT (S1, S2, and S3) corresponds to time points 14 days and 6 weeks (4,160 nuclei); and Persistent injured_PT (PT2) corresponds to time points 14 days and 6 weeks (1,078 nuclei). S1, S2, and S3 were classified as normal and recovered based on the upregulation of Lrp2, Slc5a2, Slc13a3, and Slc16a9 compared with the other clusters, while PT0, PT1, PT2, and PT3 were classified as early or persistent injured clusters based on the downregulation of Lrp2, Slc5a2, Slc13a3, and Slc16a9 and the upregulation of Vcam1 and Havcr1. FindAllMarkers function from Seurat was used to define the markers associated with the clusters. Normal_PT contains cells from the 2 data sets, while the other clusters include only cells from Kirita et al.’s data set (73). We decided to apply this gating strategy, aiming to have a clear signal and avoid variability due to experimental variability. Indeed, the level of the damage and time points may influence the kinetics of injury and recovery. Afterward, MAGIC was applied to the PT clusters extracted composed of Normal_PT, Early injured_PT, Recovered_PT, and Persistent injured_PT, and for data visualization we used RunUMAP, Featureplot, and Dotplot from Seurat.

### Cells

Normal human renal epithelial cells of proximal origin (HK2) were purchased from ATCC/LGC (lot number 60352186), then cultured according to a previously published method (76). HK2 is a cell line derived from primary PTCs. HK2 cells were cultured in DMEM containing 5 μg/mL insulin, 10 μg/mL human apotransferrin, 500 ng/mL hydrocortisone, 10 ng/mL epithelial growth factor, 6.5 ng/mL triiodothyronine, 5 ng/mL sodium selenite, 1% fetal calf serum, 25 IU/mL penicillin, 25 μg/mL streptomycin, and 10 mM HEPES buffer. These cell lines are Mycoplasm free (Mycoalert Mycoplasma Detection Kit, Lonza). Tun, Tg, DTT, BFA, and etoposide were purchased from MilliporeSigma.

### RNA extraction and RT-qPCR

Total RNA was extracted using the RNeasy Mini Kit (QIAGEN) according to the manufacturer’s protocol. Transcript expression levels were quantified through SYBR Green RT-qPCR using the QuantStudio 7 Flex Real-Time PCR System (Applied Biosystems). Vehicle-treated samples were used as controls, and the fold-changes for each tested gene were normalized to the ribosomal protein L13A (RPL13A) housekeeping gene. The relative expression levels were calculated using the 2^-ΔΔCT^ method (77). Primer sequences are listed in Supplemental Table 4.

### Protein extraction and immunoblotting

Cells were washed in PBS and incubated for 30 minutes at 4°C and in mPER lysis buffer (Thermo Fisher Scientific) with protease (Halt Protease Inhibitor Cocktail 100×, Thermo Fisher Scientific) and phosphatase inhibitors (Halt Phosphatase Inhibitor Cocktail 100×, Thermo Fisher Scientific). Extracts were centrifuged at 14,000*g* for 15 minutes at 4°C. Protein concentrations in the supernatant were measured by using a Pierce BCA Protein Assay Kit (Thermo Fisher Scientific) and Tecan Safire plate reader. Protein extracts (25 μg) were resolved by 4% to 12% SDS-PAGE (Invitrogen) and transferred to nitrocellulose membranes (iBlot, Invitrogen). Membranes were blocked with SEABLOCK blocking buffer (Thermo Fisher Scientific) for 1 hour at room temperature and then incubated overnight at 4°C with primary antibody diluted in blocking buffer. Primary antibodies were CPT2 (catalog ab181114, Abcam), SNAI1 (catalog 3879, Cell Signaling Technology), E-cadherin (catalog 610182, BD Transduction Laboratories), PARP (catalog 9532, Cell Signaling Technology), HSPA5 (binding immunoglobulin protein, BiP; GRP78, sc-1050, Santa Cruz Biotechnology), IRE1α (catalog 3294, Cell Signaling Technology), and tubulin (catalog T9026, MilliporeSigma). After washings in PBS-Tween buffer, membranes were incubated with secondary antibody coupled to fluorophores, either IRDye (R800 catalog 926-32221 and M800 catalog 926-32210, LI-COR Biosciences) or Alexa Fluor 680 (G680 catalog A21084 and R680 catalog A32734, Invitrogen). Infrared signal of membranes was revealed using an Odyssey detection system (LI-COR Biosciences).

### Immunohistochemistry

Kidney slices were fixed in alcohol–formalin–acetic acid, dehydrated with ethanol and xylene, embedded in paraffin, and cut into 3 μm sections. Samples were then deparaffinized, rehydrated, and heated for 20 minutes at 97°C in citrate buffer. Endogenous peroxidase was inactivated by incubation for 10 minutes at room temperature in 0.3% H_2_O_2_. Sections were incubated with PBS containing 1:50 anti-Hspa5 (catalog sc-1050, Santa Cruz Biotechnology) and anti-Cpt2 (catalog ab181114, Abcam). Next, sections were incubated with anti-rabbit or anti-goat antibodies conjugated with peroxidase-labeled polymer [Dako, Agilent Technologies, EnVision+ Dual Link System-HRP (DAB+) catalog K4065], then visualized with a peroxidase kit (Dako). Finally, the tissue sections were counterstained with hematoxylin.

### Oil Red O staining

Optimal cutting temperature compound–embedded (Agar Scientific), frozen kidney biopsy sections and paraformaldehyde-fixed cells were used for Oil Red O staining. Slides were washed with PBS and fixed with 4% paraformaldehyde (PFA) for 15 minutes at room temperature. After removing the 4% PFA, slides were washed twice with PBS. Samples were labeled with Oil Red O solution for 20 minutes at room temperature. After the Oil Red O staining, slides were washed twice with PBS before picture acquisition with a microscope (NIKON Eclipse Ti).

### siRNA transfections

The transient inactivation of CPT2 was achieved using siRNAs designed by and obtained from QIAGEN and transfected using HiPerFect (QIAGEN) according to the manufacturer’s protocol. Two siRNAs directed against the same target were transfected: Hs CPT2 1 Flexi Tube siRNA si00353269 and Hs CPT2 5 Flexi Tube siRNA si03039540. AllStars Negative Control siRNA (5′-AACGAUGACACGAACACACTT-3′) has no homology to any known mammalian gene, and validation was performed using Affymetrix GeneChip arrays and a variety of cell-based assays to ensure minimal nonspecific effects on gene expression and phenotype. Cells were incubated with siRNA for 24 hours before conducting the experiments.

### Shotgun lipidomics

For lipidomics analysis, 100,000 HK2 cells were spiked with 3.23 μL of internal standard lipid mixture containing 500 pmol of Chol-d6, 100 pmol of Chol-16:0-d7, 100 pmol of DAG 17:0-17:0, 50 pmol of triglycerides 17:0-17:0-17:0, 100 pmol of SM 18:1;2-12:0, 30 pmol of Cer 18:1;2-12:0, 30 pmol of GalCer 18:1;2-12:0, 50 pmol of LacCer 18:1;2-12:0, 300 pmol of PC 17:0-17:0, 50 pmol of PE 17:0-17:0, 30 pmol of PI 16:0-16:0, 50 pmol of PS 17:0-17:0, 30 pmol of PG 17:0-17:0, 30 pmol of PA 17:0-17:0, 25 pmol of Gb3 18:1;2-17:0, 25 pmol of GM3 18:1;2-18:0-d5, 25 pmol of GM2 18:1;2-18:0-d9, 25 pmol of GM1 18:1;2-18:0-d5, 30 pmol of lysophosphatidic acid (LPA) 17:0, 30 pmol of lysophosphatidylcholine (LPC) 12:0, 30 pmol of lysophosphatidylethanolamine (LPE) 17:1, and 30 pmol of LPS 17:1 and subjected to lipid extraction at 4°C, as described elsewhere (78). Briefly, the sample was dissolved in 200 μL of 155 mM ammonium bicarbonate and then extracted with 1 mL of chloroform-methanol (10:1) for 2 hours. The lower organic phase was collected, and the aqueous phase was re-extracted with 1 mL of chloroform-methanol (2:1) for 1 hour. The lower organic phase was collected and evaporated in a SpeedVac vacuum concentrator (Thermo Fisher Scientific). Lipid extracts were dissolved in 100 μL of infusion mixture consisting of 7.5 mM ammonium acetate dissolved in propanol/chloroform/methanol (4:1:2 vol/vol). Samples were analyzed by direct infusion in a Q Exactive mass spectrometer (Thermo Fisher Scientific) equipped with a TriVersa NanoMate ion source (Advion Biosciences). A total of 5 μL of sample was infused with gas pressure and voltage set to 1.25 psi and 0.95 kV, respectively.

Triglycerides and CE-Chol were detected in the 10:1 extract, by positive ion mode Fourier transform mass spectrometry (FTMS) as ammonium adducts by scanning *m/z* = 580–1,000 Da, at R_m/z = 200_ = 280,000, with lock mass activated at a common background (*m/z* = 680.48022) for 30 seconds. Every scan is the average of 2 micro-scans, automatic gain control (AGC) was set to 1 × 10^6^, and maximum ion injection time (IT) was set to 50 ms. For FA profiling of triglycerides, a parallel reaction monitoring was performed with an inclusion list of *m/z* = 580–1,000 Da, at normalized collision energy of 20 and R_m/z = 200_ = 17,500, for 30 seconds. Every scan is the average of 2 micro-scans, AGC was set to 1 × 10^6^, and maximum ion IT was set to 64 ms. LPC and lysophosphatidylcholine plasmalogen were detected as acetate adducts, and LPE and lysophosphatidylethanolamine plasmalogen were detected as deprotonated adducts in the 10:1 extract, by negative ion mode FTMS by scanning *m/z* = 4,200–1,050 Da, at R_m/z = 200_ = 280,000, with lock mass activated at a common background (*m/z* = 529.46262) for 30 seconds. Every scan is the average of 2 micro-scans, AGC was set to 1 × 10^6^, and maximum ion IT was set to 50 ms. LPA, lysophosphatidylinositol, and LPS were detected in the 2:1 extract, by negative ion mode FTMS as deprotonated ions by scanning *m/z* = 400–1,100 Da, at R_m/z = 200_ = 280,000, with lock mass activated at a common background (*m/z* = 529.46262) for 30 seconds. Every scan is the average of 2 micro-scans, AGC was set to 1 × 10^6^, and maximum ion IT was set to 50 ms. All data were acquired in centroid mode. All lipidomics data were analyzed with the lipid identification software LipidXplorer (79). Tolerance for MS and identification was set to 2 ppm. Data postprocessing and normalization to internal standards were done manually in Microsoft Excel. For the sake of simplicity, only the pertinent data are displayed (LysoPLs, triglycerides, and CE-Chol) and normalized to the total lipid identified.

### Mitochondrial activity measurements

For measurements of OCR by Seahorse XFe96 Analyzer (Agilent Technologies), HK2 cells were seeded at a density of 6 × 10^4^ cells per well in a collagen-coated XFe96 cell culture microplate (Agilent Technologies). Twenty-four hours postplating cells were infected with relevant drugs, or 48 hours after siCPT2 transfection, and mitochondrial activity was assessed. Before measurement cells were balanced for 1 hour in unbuffered XF assay media (Agilent Technologies) supplemented for OCR analysis with 2 mM glutamine, 10 mM glucose, and 1 mM sodium pyruvate. For OCR measurements, compounds were injected during the assay at the following final concentrations: oligomycin (ATP synthase inhibitor to measure respiration associated with cellular ATP production, 1 μM), FCCP (uncoupling agent to measure the maximal respiration capacity; 1 μM), and rotenone and antimycin A (electron transport chain inhibitors to measure the nonmitochondrial respiration; 1 μM each).

### Data availability

Expression profiling by high-throughput sequencing for human kidney transplant biopsies is available at GEO (GSE126805).

Expression profiling by high-throughput sequencing for mouse IRI (bulk RNA-Seq) is available at GEO (GSE98622). snRNA-Seq data of mouse IRI are available at GEO (GSE151167 and GSE139107). Expression profiling by array for human diabetic kidneys is available at GEO (GSE30122). Expression profiling by high-throughput sequencing for UmodC147W/+ kidneys and UMOD-expressing epithelium are available at GEO (GSE1102566).

Transcriptomic data for human CKD patients and healthy donors are available at Nephroseq at the data set titled “Ju CKD TubInt.”

Raw data of metabolomic and lipidomic analyses and clinical data are available from the corresponding authors upon reasonable request.

### Statistics

Graphs and statistical analyses were generated using GraphPad Prism 9 Software. Metabolomics analyses were performed using MetaboAnalyst 5.0. Data are reported as mean ± standard deviation. Number of samples assayed in each experiment is indicated in the figure legends. Unpaired 2-sample Student’s *t* tests were used to determine a significant difference between 2 groups with *n* > 5. Student’s *t* tests were 2 tailed. One-way or 2-way ANOVA was performed when more than 2 groups were compared. Dunnett’s multiple comparisons test was used for multiple comparisons. For small-size samples, Mann-Whitney or Kruskal-Wallis test was performed to compare 2 or more groups, respectively. For the prediction of binary ci 12 months after transplantation, we trained a logistic regression model in leave-one-out cross-validation setting. We pooled together all the predictions, we produced the receiver operating characteristic curve, and we calculated its underlying area (AUROC). This analysis was performed in R. *P* < 0.05 was considered to represent a statistically significant difference.

### Study approval

#### Leuven KTR cohort for kidney allograft RNA-Seq.

Patients were enrolled at the University Hospitals of Leuven (23). Participants provided written informed consent, and the study was approved by the Ethical Review Board of the University Hospitals of Leuven (S53364 and S59572, Leuven, Belgium).

#### Necker KTR cohort.

This study was approved by the Ethics Committee of Ile-de-France XI (approval 13016, Paris, France), and all the participating patients provided written informed consent.

#### Animal experiments.

Regarding the tunicamycin model, experimentation was performed with the approval of the Institutional Animal Care and Use Committee of the Paris Descartes University (Paris, France), protocol B2013-59. Experiments were conducted according to French veterinary guidelines and those formulated by the European Commission for experimental animal use (L358–86/609/EEC). We retrieved data from previously published, public data sets for other animal experiments.

## Author contributions

AR, HL, VP, and YB performed experiments. NP analyzed data sets. IN performed metabolomic experiments. JLS performed lipidomic experiments. DM performed bioinformatic analyses. MR performed Oil Red O staining. MN and PEC provided RNA-Seq data from kidney allograft recipients and performed analyses. DA generated Necker KTR data sets and urine biosample collection. PEC and AR conducted snRNA-Seq experiments and analyses. NP conceived and designed the project and wrote the paper.

## Supplementary Material

Supplemental data

## Figures and Tables

**Table 1:**
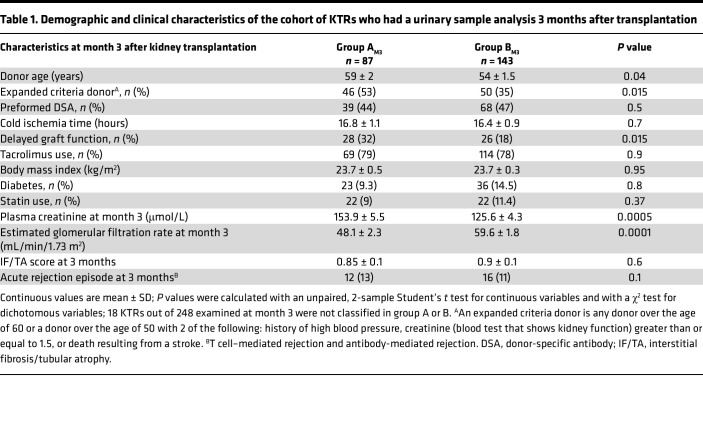
Demographic and clinical characteristics of the cohort of KTRs who had a urinary sample analysis 3 months after transplantation

## Version 1 08/23/2022

In-Press Preview

## Version 2 09/22/2022

Electronic publication
