# Supplementary material for: Impaired fatty acid metabolism perpetuates lipotoxicity along the transition to chronic kidney injury
Source: JCI Insight. 2022 Sep 22;7(18):e161783. doi: 10.1172/jci.insight.161783 (PMC9675570; doi:10.1172/jci.insight.161783)
Supplement: Supplemental data [file jciinsight-7-161783-s056.pdf]

Supplementary Table 1. Demographic and clinical characteristics of the cohort of 272 kidney transplant recipients who had a urinary sample metabolic profiling 3 months and/or 12 months after transplantation.

**Characteristics**	**Entire cohort** (n=272)
Age (years)	51.1±15.2
Male sex-n (%)	159 (58)
Cause of ESRD-n (%)	
• GN	55 (20)
• Diabetes	38 (14)
• Cystic/hereditary	54 (20)
• Secondary GN	23 (8.5)
• Hypertension	18 (6)
• Interstitial nephritis	18 (6)
• Miscellaneous	10 (3)
• Uncertain	56 (20)
Donor age (years)	54 (19)
Living donor-n (%)	63 (23)
Expanded criteria donor-n (%)	109 (40)
Retransplantation-n (%)	40 (15)
Preformed DSA-n (%)	124 (45)
Cold ischemia time (hours)	16.5±10
Delayed graft function-n (%)	66 (24)
Plasma creatinine at day 10 (μmol/L)	242±261

ESRD: End Stage Renal Disease

GN: glomerulonephritis

DSA: donor specific antibodies

Supplementary Table 2. Multivariate analysis of parameters associated with the group A

Variable	Estimate	95% CI	P value
Intercept	1.209	0.2 to 2.3	0.02
Donor age	-0.005	-0.03 to 0.015	0.6
DGF [Yes]	-0.7	-1.35 to -0.07	0.03
ECD [Yes]	-0.5	-1.3 to 0.3	0.2

DGF denotes delayed graft function and refers to the acute kidney injury that occurs in the first week of kidney transplantation, which necessitates dialysis intervention.
ECD denotes extended criteria donor and refers to any donor over the age of 60, or a donor over the age of 50 with two of the following: a history of high blood pressure, a creatinine greater than or equal to 1.5 mg/dl, or death resulting from a stroke.

Supplementary Table 3. Demographic and clinical characteristics of the cohort of kidney transplant recipients according to the change of community between 3 and 12 months after transplantation.

**Characteristic of KTR according to community at M3 and M12**	**Community A_{M3}>A_{M12}** n= 33	**Community A_{M3}>B_{M12}** n= 47	**Community B_{M3}>B_{M12}** n= 104	***P*-value**
Donor Age (years)	59±2	60±1.5	53.3±1.2	0.002
Living donor-n (%)	7 (20)	4 (8)	30 (29)	<0.0001
Expanded criteria donor-n (%)	17.5 (52)	30.5 (65)	35.5 (33)	<0.0001
Cold ischemia time (hours)	933±63	1028±53	974±43	0.09
Delayed graft function-n (%)	15 (44)	13 (27)	17 (16)	<0.0001
Tacrolimus use-n (%)	27 (79)	36 (76)	85.5 (80)	0.8
eGFR at month 3 (ml/min/1.73m^2)	44±2.5	50±2	61±1.5	<0.0001
eGFR at month 12 (ml/min/1.73m^2)	43.5±2.7	51±2.2	60.5±1.5	<0.0001

KTR : Kidney transplant recipient

eGFR : estimated glomerular filtration rate

Supplementary Table 4. RT-qPCR primers list

huHSPA5-Fwd GGT GAA AGA CCC CTG ACA AA
huHSPA5-Rev GTC AGG CGA TTC TGG TCA TT

huCPT2-Fwd AGC CTC TCT TGA ATG ATG GCC
huCPT2-Rev GAT AGG TAC ATA TCA AAC CAG GG

huCPT1a-Fwd TCC AGT TGG CTT ATC GTG GTG
huCPT1a-Rev CTA ACG AGG GGT CGA TCT TGG

huSNAIL-Fwd CAC TAT GCC GCGC TCT TTC
huSNAIL-Rev GCT GGA AGG TAA ACT CTG GAT TAG A

huFibronectin-Fwd ACT CCC TTT TCT CCT CTT GTG
huFibronectin-Rev CGC TGT TGT TTG TGA AGT AGAC

hu-Vimentin-Fwd CCC TCG TTC GCC TCT TCT C
hu-Vimentin-Rev TTG GTG CGG GTG TTC TTG

hu-IL6-Fwd CCA GGA GAA GAT TCC AAA GAT GTA
hu-IL6-Rev CGT CGA GGA TGT ACC GAA TTT

huMCP1-Fwd CCC CAG TCA CCT GCT GTT AT
huMCP1-Rev TGG AAT CCT GAA CCC ACT TC

huIL8-Fwd CAG GAA TTG AAT GGG TTT GC
huIL8-Rev AAA CCA AGG CAC AGT GGA AC

huEcadherin-Fwd TAC GCC TGG GAC TCC ACC TA
huEcadherin-Rev CCA GAA ACG GAG GCC TGA T

husXBP1-Fwd TGC TGA GTC CGC AGC AGG TG
husXBP1-Rev GCT GGC AGG CTC TGG GGA AG

huXBP1-Fwd AAA CAG AGT AGC AGC TCA GAC TGC
huXBP1-Rev TCC TTC TGG GTA GAC CTC TGG GAG

huEDEM1-Fwd CAA GTG TGG GTA CGC CAC G
huEDEM1-Rev AAA GAA GCT CTC CAT CCG GTC

huERDJ4-Fwd CCA AAA TCG GCA TCA GAG CG
huERDJ4-Rev ATT TTG CTT CAG CAT CCG GG

huGADD34-Fwd AGG AGG CTG AAG ACA GTG GA
huGADD34-Rev GGC CAT CTG CAA ATT GAC TT

huRPL13a-Fwd CCT GGA GGA GAA GAG GAA AGA GA
huRPL13a-Rev GAG GAC CTC TGT GTA TTT GTC AA

mHspa5-Fwd CAC CAG GAT GCG GAC ATT GA
mHspa5-Rev AGG GCC TCC ACT TCC ATA GA

mCpt2-Fwd TGT GAG CGG AAG ATC CCA AC
mCpt2-Rev GCT TTC CAA CCC GAT CTC CT

mRpl13a-Fwd CCC TAT GAC AAG AAA AAG CGG A
mRpl13a-Rev TTT CCT TCC GTT TCT CCT CCA G

mTbp-Fwd CAA ACC CAG AAT TGT TCT CCT T
mTbp-Rev ATG TGG TCT TCC TGA ATC CCT

SUPPLEMENTARY FIGURE 1

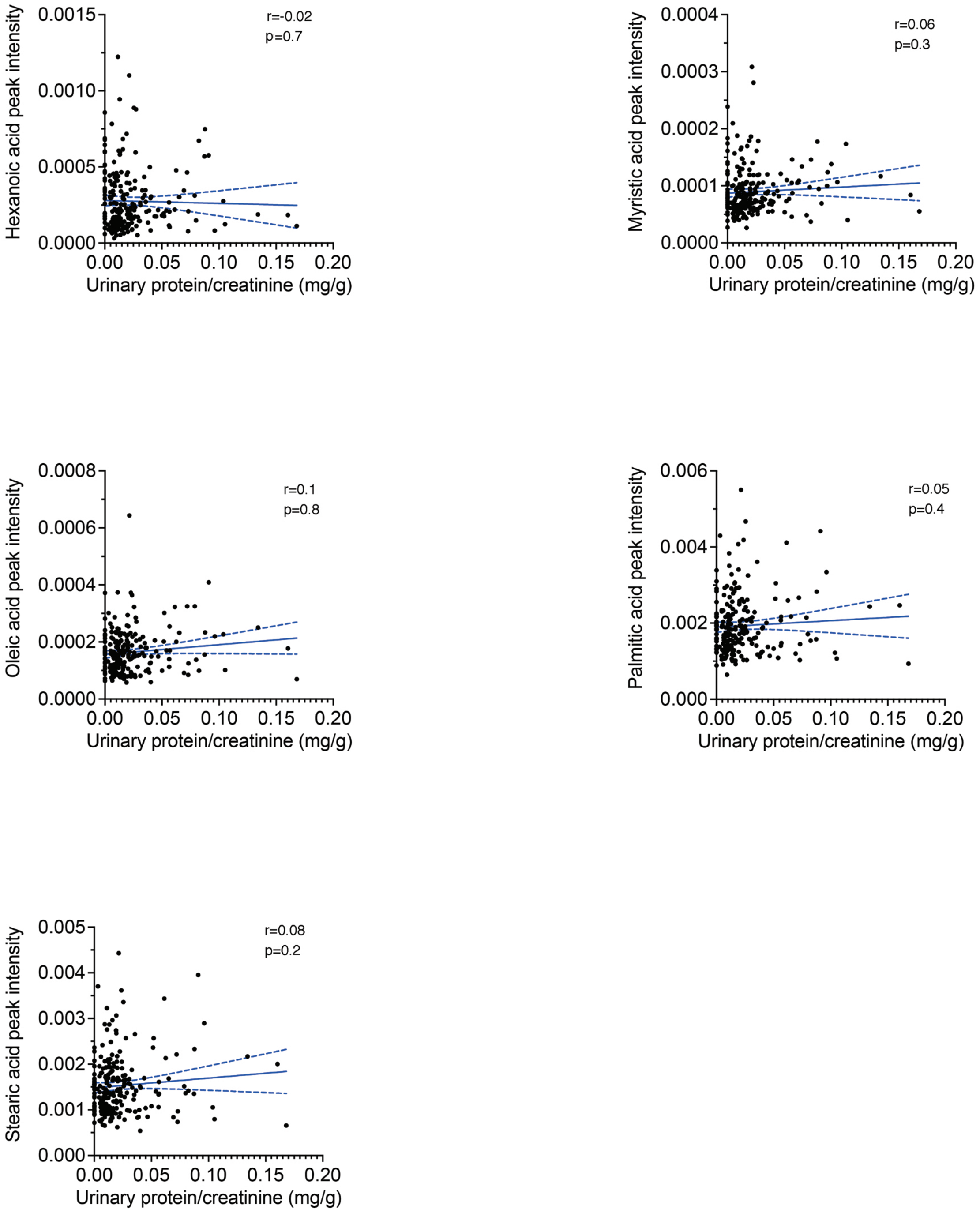

SUPPLEMENTARY FIGURE 2

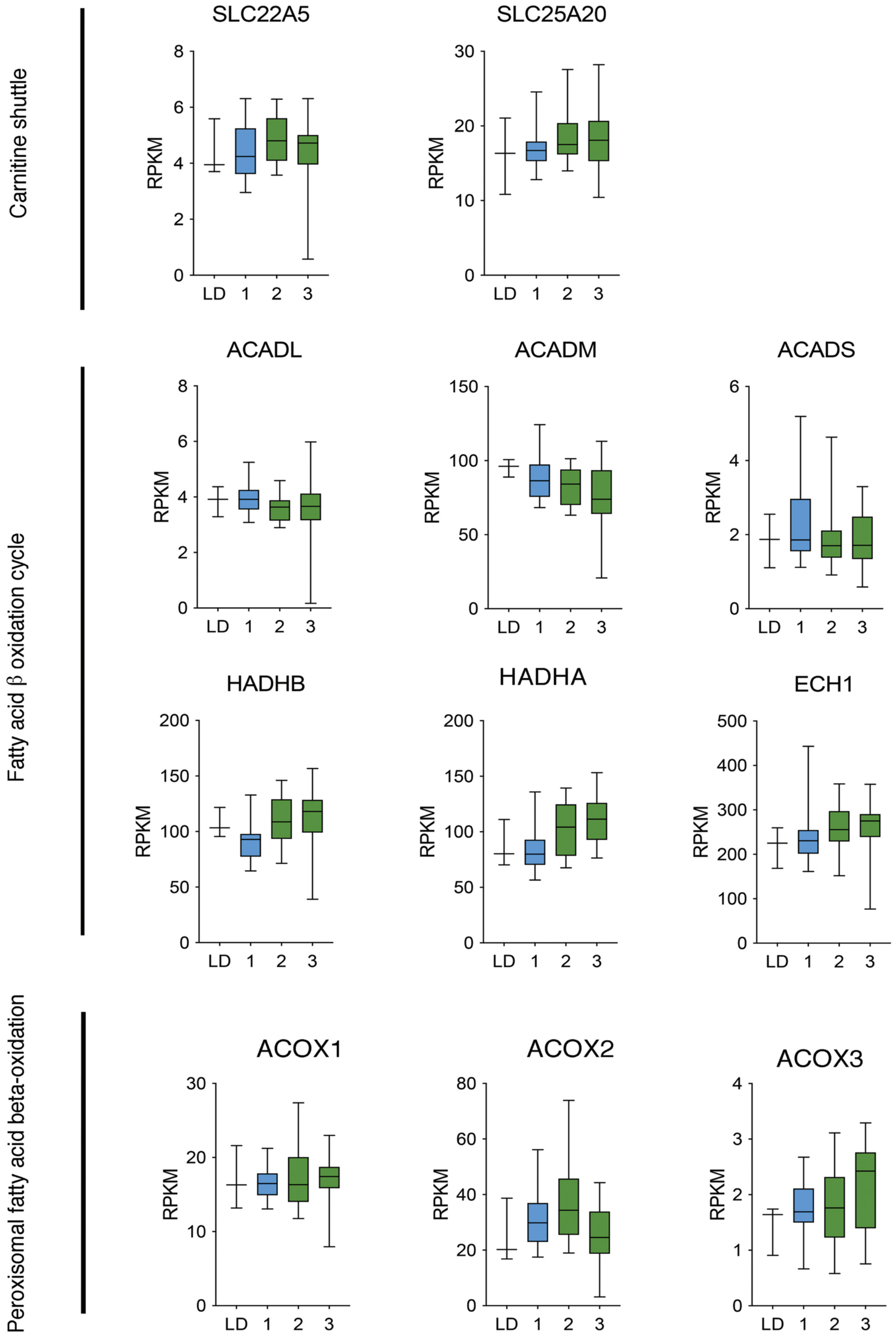

SUPPLEMENTARY FIGURE 3

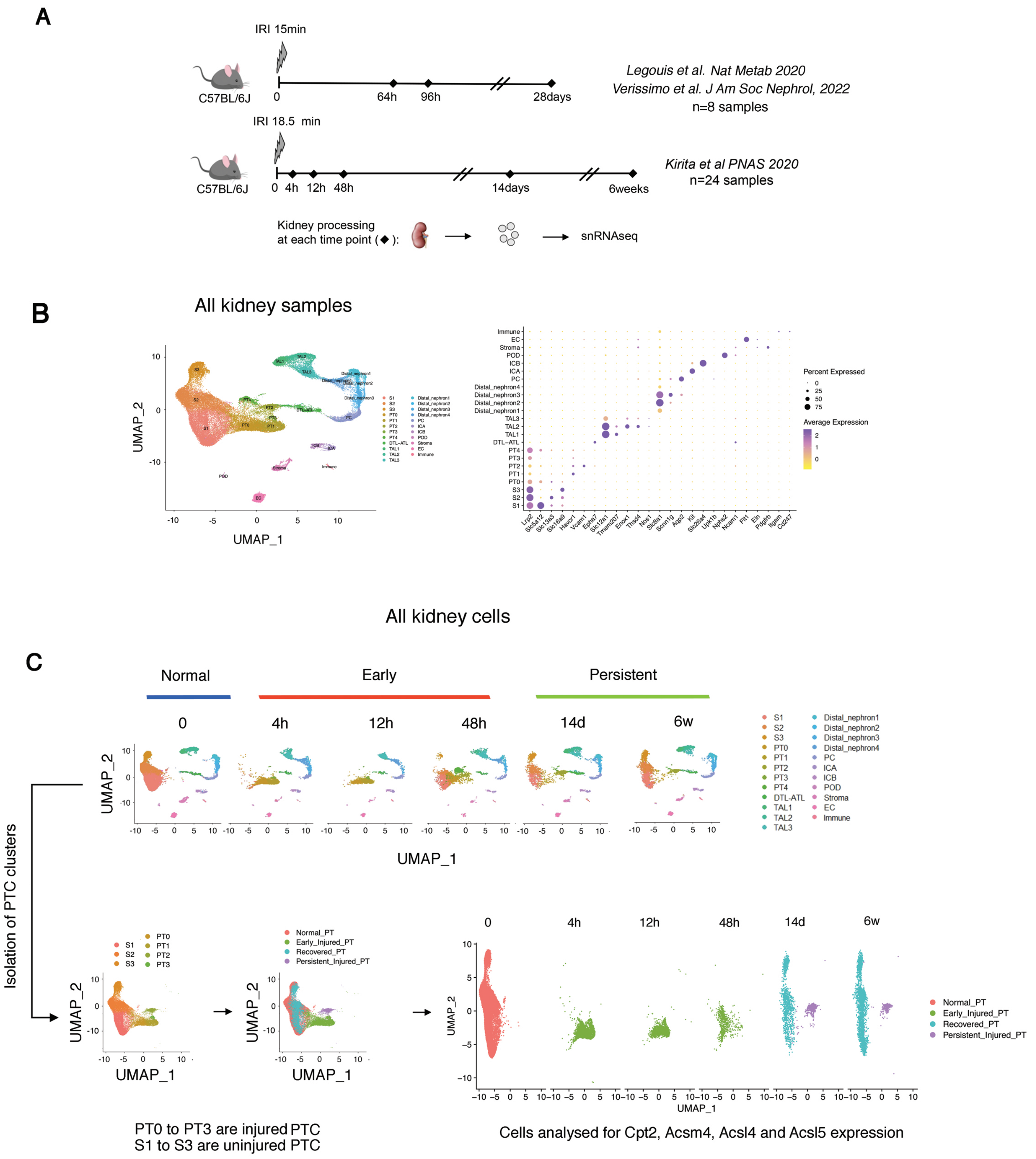

SUPPLEMENTARY FIGURE 4

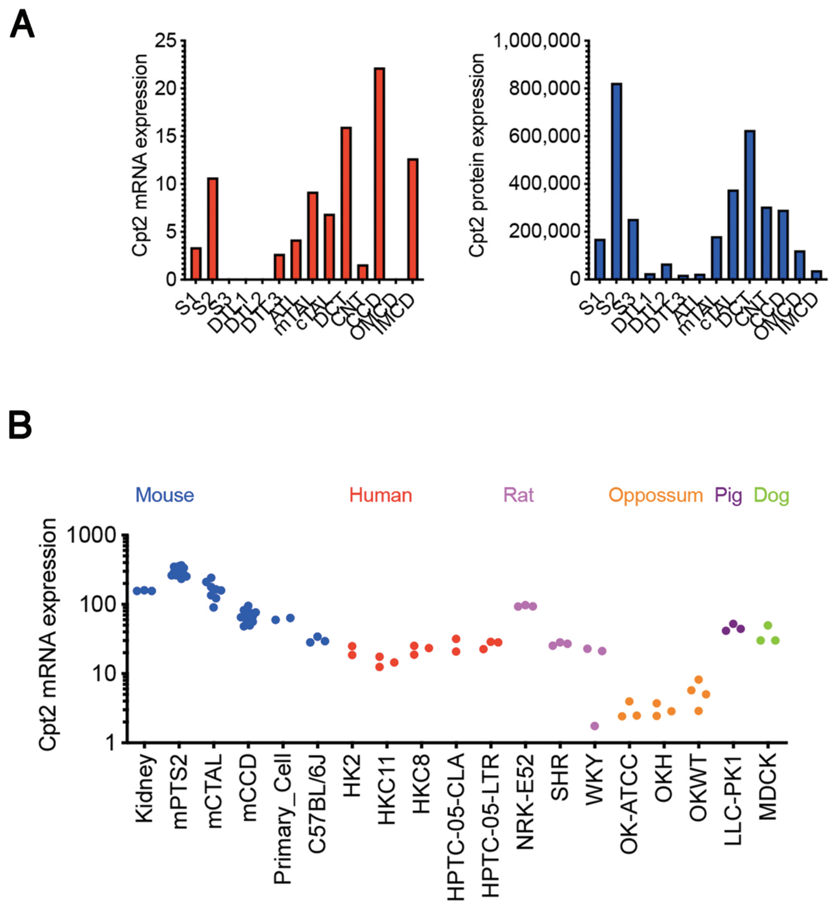

SUPPLEMENTARY FIGURE 5

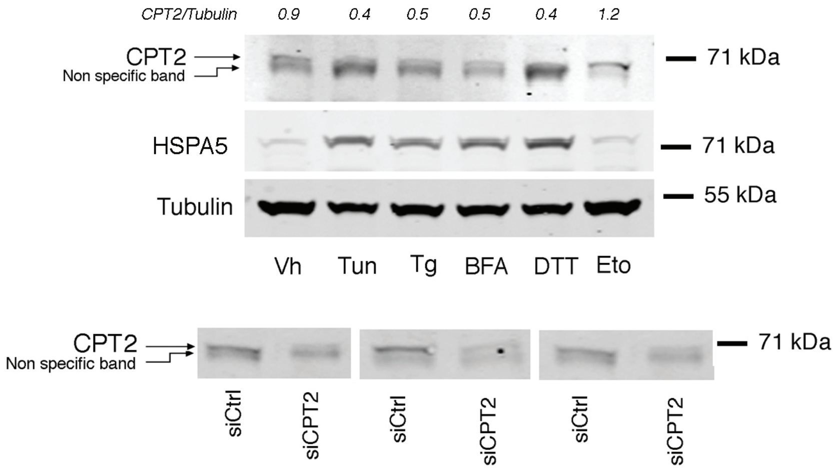

SUPPLEMENTARY FIGURE 6

A

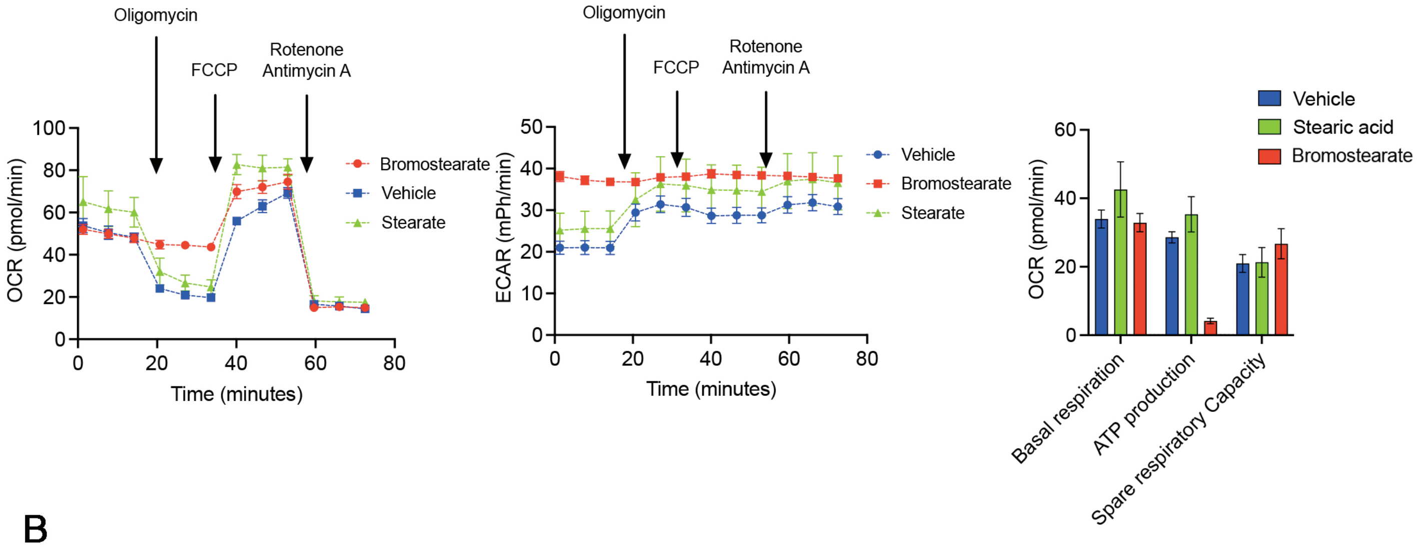

B

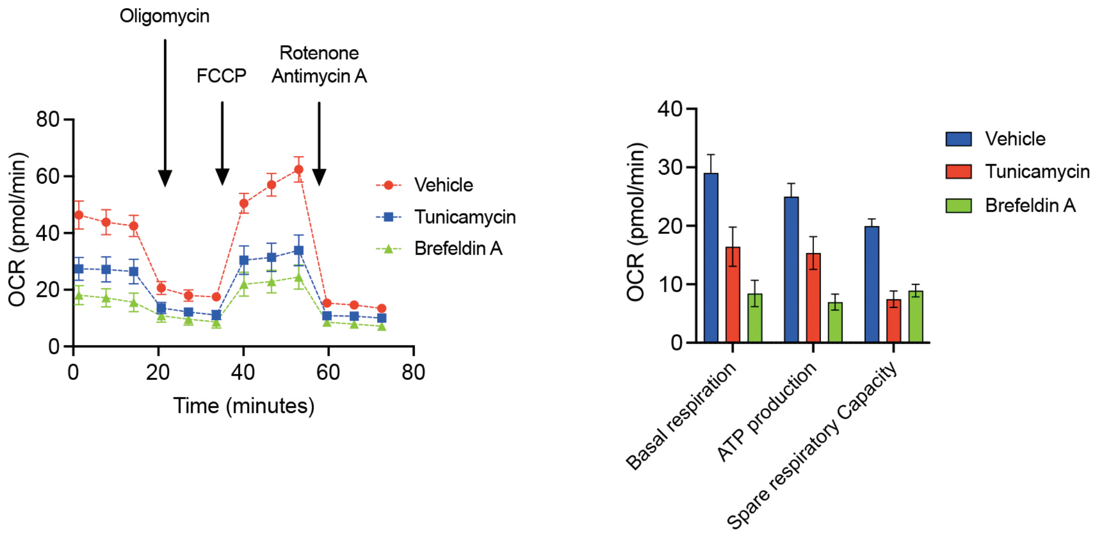

SUPPLEMENTARY FIGURE 7

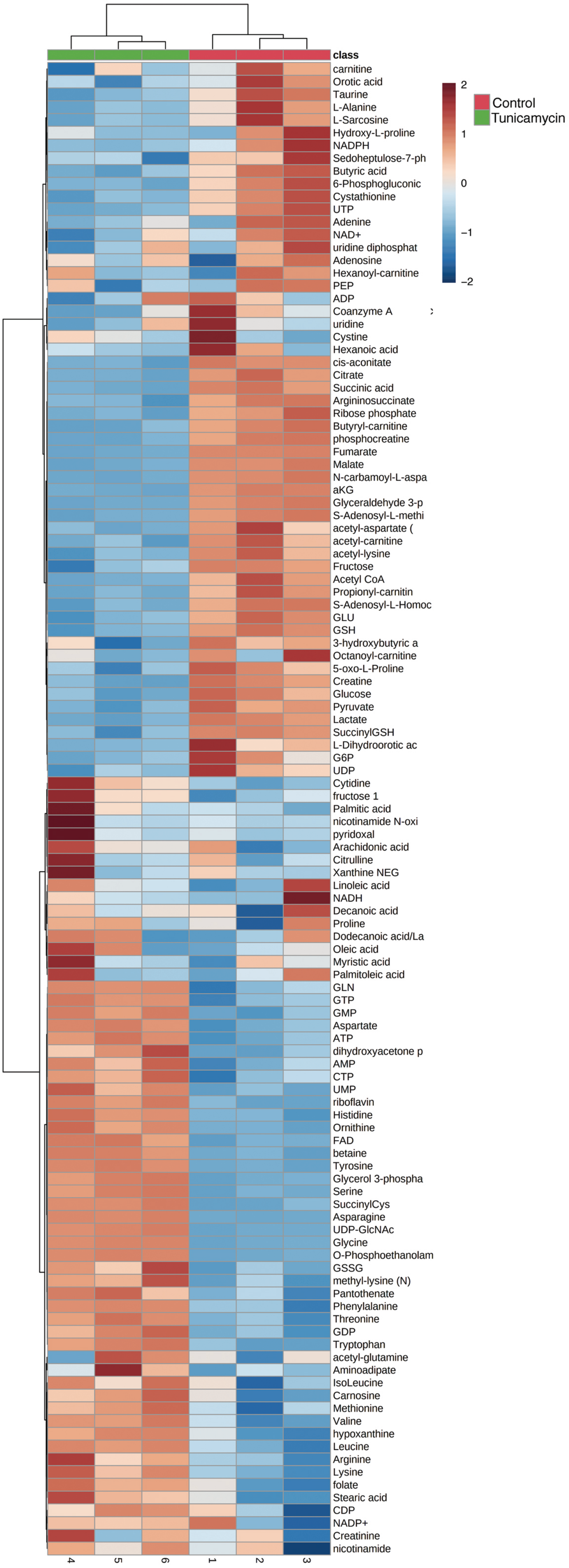

SUPPLEMENTARY FIGURE 8

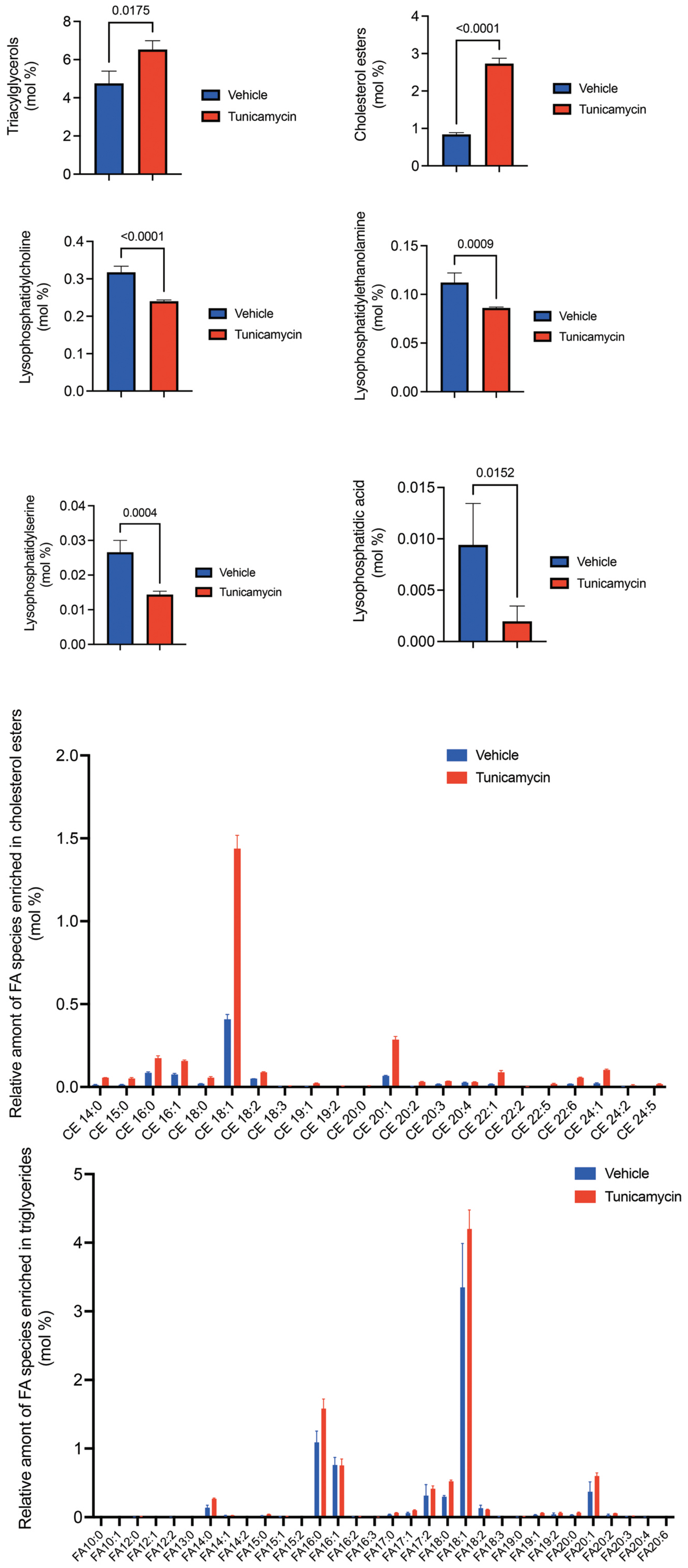

SUPPLEMENTARY FIGURE 9

A

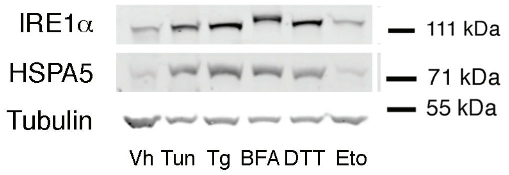

B

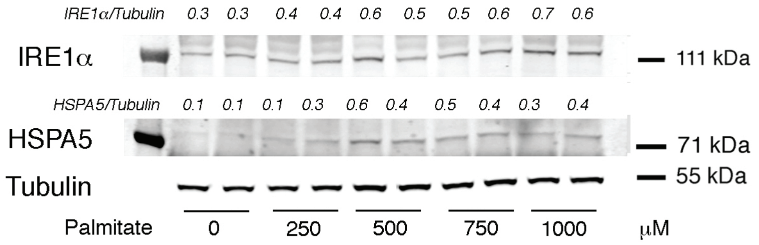

SUPPLEMENTARY FIGURES LEGENDS

SUPPLEMENTARY FIGURE 1

Correlation between urine protein levels (mg/g creatinine) and FA urine levels (standardized peak intensity) measured in 248 KTR 3 months after transplantation. P values were calculated with a Student's T test. The dashed lines represent the 95% confidence intervals.

SUPPLEMENTARY FIGURE 2

Expression of various genes involved in FAO measured by RNA-sequencing (RNA-seq) of mRNA isolated from whole-kidneys in the group of 42 kidney transplant recipients who recovered or progressed to fibrosis according to a computational model described, which identified 2 main transcriptional trajectories leading to kidney recovery or to sustained injury with associated fibrosis and renal dysfunction. p values computed using Student's t-test did not reach statistical significance.

SUPPLEMENTARY FIGURE 3.

A. Schematic of the experimental strategy of single nucleus RNA sequencing (snRNAseq) data. Ischemia reperfusion injury (IRI) was induced for 15 or 18.5 minutes and the kidneys collected at the depicted time points respectively. At each time point kidneys were processed and analysed by snRNAseq.

B. (*Left panel*) Uniform manifold approximation and projection (UMAP) plot of kidney samples analysed by snRNAseq and integrated (n=32; 60'360 renal nuclei) identifies the different cellular components of the kidney. Proximal Tubule Segment S1, S2, S3; new (injured) proximal tubule clusters (PT0, PT1, PT2, PT3, PT4); DTL, descending limb of loop of Henle; ATL, thin ascending limb of loop of Henle; TAL, thick ascending limb of loop of Henle (TAL1, TAL2, TAL3); POD, podocytes; Distal nephron (cluster 1, 2, 3); ICA, type A intercalated cells of collecting duct; ICB, type B intercalated cells of collecting duct; PC, principle cells; EC, endothelial cells, Immune and Stroma cells. (*Right panel*) Dot plot displaying gene expression for the renal cell types. See Supplementary Information for the markers characterizing each cluster.

C. (*Upper panel*) UMAP of kidney samples at the selected time points (0, 4h, 12h, 48h, 14d, 6w) including n= 51'787 renal nuclei. (*lower panel*) UMAP of the PTC cells annotated based on injury and differentiation markers at different time points (n=26; 27'148 renal nuclei).

SUPPLEMENTARY FIGURE 4.

A. Histograms representing the expression of Cpt2 transcripts and proteins in rat tubules. Data are from public repositories pertaining to the transcriptome (NCBI accession GSE56743 and the publicly accessible webpage (https://helixweb.nih.gov/ESBL/Database/NephronRNAseq/index.html). Proximal Tubule Segment S1, S2, S3; DTL, descending limb of loop of Henle; ATL, thin ascending limb of loop of Henle; TAL, thick ascending limb of loop of Henle; DCT, distal convoluted tubule; CNT, connecting tubule; CCD: cortical collecting duct; OMCD: outer meddulary Collecting duct; IMCD: inner meddulary collecting duct.

B. Histograms representing the expression of CPT2 transcripts in various human and non-human cell lines. Data are from public repositories pertaining to the transcriptome of these cell lines (https://esbl.nhlbi.nih.gov/JBrowse/KCT/).

SUPPLEMENTARY FIGURE 5

Immunoblot representing (upper panel) Cpt2, Hspa5 and tubulin protein expression in HK2 24 h after incubation either with 250 μg/ml tunicamycin (Tun), 5 μg/mL brefeldin A (BFA), 0.25 μM thaspigargin (Tg), 1 μM dithiotreitol (DTT), 100 μM etoposide (Eto) or DMSO for 24 h, and (lower panel) 48 hours after transfection of siRNA targeting CPT2. The immunoblot shown is representative of 3 independent experiments. The extinction of the upper band, but not the lower band, under siCPT2 transfection highlights the specificity for CTP2 of the upper band.

SUPPLEMENTARY FIGURE 6

A. The OCR and ECAR measured by SeaHorse Bioanalyzer in HK-2 cells in response to incubation either with DMSO, 500 µM stearate or 20 µM bromostearate for 24h. or 250 µg/ml tunicamycin for 24h. Dashed lines indicate the time of the addition of each reagent. Quantification of basal respiration, ATP production and maximal respiratory capacity are shown on the graphs (right panel).

B. The OCR measured by SeaHorse Bioanalyzer in HK-2 cells in response to incubation either with DMSO, 5 µg/mL brefeldin A or 250 µg/ml tunicamycin for 24h. Dashed lines indicate the time of the addition of each reagent. Quantification of basal respiration, ATP production and maximal respiratory capacity are shown on the graphs (right panel). Data are means ± SEM.

SUPPLEMENTARY FIGURE 7

Hierarchical clustering (Ward method) of HK2 cells incubated with 250 µg/ml tunicamycin (Tun) for 24 h of DMSO (n=3 per condition). Each line represents the relative composition of metabolites identified by liquid chromatography coupled with tandem mass spectrometry using SeQuant ZIC-pHilic columns. Each ~~line~~ column represents a condition or a replicate. Samples were normalized by sum and data were autoscaled.

SUPPLEMENTARY FIGURE 8

Mass spectrometry-based lipid analysis of HK-2 cells incubated with 250 µg/ml tunicamycin (Tun) for 24 h of DMSO (n=3 per condition). Lipids class such as triacylglycerols (TAG), Cholesterol esters (CE-chol), and lysophospholpids (LysoPL) were selected. The relative enrichment in FA with their chain length in CE-Chol and TAG are represented.

SUPPLEMENTARY FIGURE 9

A. Immunoblot representing (upper panel) IRE1α Hspa5 and tubulin protein expression in HK2 cells 24 h after incubation either with 250 µg/ml tunicamycin (Tun), 5 µg/mL brefeldin A (BFA), 0.25 µM thaspigargin (Tg), 1 µM dithiotreitol (DTT), 100 µM etoposide (Eto) or DMSO for 24 h, to determine the IRE1α expression profile during the unfolded protein response.

B. Immunoblot representing IRE1α HSPA5 and tubulin protein expression in HK2 cells 24 h after incubation with increasing concentrations of palmitate.
